# Unsupervised design and geometry optimization of high-sensitivity ring-resonator-based sensors

**DOI:** 10.1038/s41598-025-03056-x

**Published:** 2025-05-23

**Authors:** Tanveerul Haq, Slawomir Koziel, Anna Pietrenko-Dabrowska

**Affiliations:** 1 Engineering Optimization & Modeling Center, Reykjavik University, 101, Reykjavik, Iceland; 2 Faculty of Electronics, Telecommunications and Informatics, Gdansk University of Technology, Gdansk, 80-233 Poland

**Keywords:** Microwave sensors, High-sensitivity sensors, Dielectric characterization, Artificial intelligence, Unsupervised design, EM-driven design, Engineering, Electrical and electronic engineering

## Abstract

In this study, we introduce a technique for unsupervised design and design automation of resonator-based microstrip sensors for dielectric material characterization. Our approach utilizes fundamental building blocks such as circular and square resonators, stubs, and slots, which can be adjusted in size and combined into intricate geometries using appropriate Boolean transformations. The sensor’s topology, including its constituent components and their dimensions, is governed by artificial intelligence (AI) techniques, specifically evolutionary algorithms, in conjunction with gradient-based optimizers. This enables not only the explicit enhancement of the circuit’s sensitivity but also ensures the attainment of the desired operating frequency. The design process is entirely driven by specifications and does not necessitate any interaction from the designer. We extensively validate our design framework by designing a range of high-performance sensors. Selected devices are experimentally validated, calibrated using inverse modeling techniques, and utilized for characterizing dielectric samples across a wide spectrum of permittivity and thickness. Moreover, comprehensive benchmarking demonstrates the superiority of AI-generated sensors over state-of-the-art designs reported in the literature.

## Introduction

Microwave sensors are highly valuable instruments in numerous industries due to their adaptability and efficacy^1^. They have made significant advancements in numerous sectors, such as aerospace^2^, agriculture^3^, defense^4^, and food processing^5^. Microwave sensors employ either resonant or non-resonant techniques based on the specific application. Non-resonant microwave sensors detect environmental changes by analyzing the reflection, absorption, or scattering of microwave signals^6^. These sensors are often used for applications requiring rapid response times, non-contact operation, and robustness. In contrast, resonant microwave sensors detect environmental changes by utilizing the resonant frequency of a cavity or structure^7^. These sensors are widely employed in applications that need accurate measurements or detection of specific materials, such as biomedical sensing to detect changes in tissue properties^8^ or electronic applications for exact material characterization^9^. The resonant approach is gaining popularity because it employs novel metamaterial (MTM) resonators to achieve high sensitivity over a broad frequency range^10^. MTM structures, such as complementary split ring resonators (CSRRs)^11^ and split ring resonators (SRRs)^12^, are frequently used in resonant microwave sensors due to their multi-functionality, compactness, cost-effectiveness, versatility, and ability to integrate with other microwave technologies.

Recently, SRRs and CSRRs-based microwave sensors have been used in various applications such as concentration measurement^13^, high-resolution imaging^14^, permittivity evaluation^15^, remote sensing^16^, surface crack detection^17^, and soil moisture monitoring^18^. Most of these sensors are susceptible to common ohmic and dielectric losses. This results in a low-quality factor, which restricts the sensitivity of these passive microwave sensors. An approach proposed to address this issue involves augmenting the resonator with amplifying circuits to compensate for power loss using a recovery mechanism^19^. This approach has the potential to enhance sensitivity and performance by reducing the impact of power loss in the resonator, but it also leads to higher power consumption, complexity, and cost. Machine-learning techniques for data processing of resonant microwave sensors have been devised to minimize power consumption and costs while improving performance. In^20^, the fuzzy neural network (FNN) method has been applied during the post-processing phase of sensing to greatly improve the resolutions of active planar ring resonators. The study in^21^ focuses on increasing the accuracy of glucose monitoring in the interstitial fluid using a super-resolution generative adversarial network (SRGAN). In^22^, machine learning algorithms, specifically classifiers and regressors, have been used to achieve the goal of developing a temperature compensation technique for microwave sensors to address the sensors’ vulnerability to ambient temperature changes. In^23^, a machine learning-aided (MLA) technique utilizing artificial neural networks (ANNs) is introduced, offering a method for directly determining the complex permittivity of both dispersive and non-dispersive materials using cost-effective measurements. Meanwhile, in^24^, an artificial intelligence (AI)-assisted approach is applied to enhance the selectivity of microwave sensors for sensing liquid mixtures. While these methods provide significant advantages in sensor data processing and precision, they do not specifically address the improvement of resonator sensitivity. Resonator sensitivity is typically influenced by factors such as the resonant frequency of microwave sensors, the electromagnetic properties of the material under test (MUT), and the interaction between the MUT and the resonator’s electromagnetic (EM) fields^25^. Interactive techniques, like parameter sweeping^26^, have been employed to develop high-sensitivity resonators in practical applications, achieving a maximum sensitivity of 8%^27^. However, this approach is computationally inefficient as it necessitates a full EM wave simulation for every combination of geometrical parameters, leading to time-consuming computations. Additionally, active involvement of the designer is required, often resulting in unsatisfactory outcomes.

As elucidated earlier, the properties of microwave sensors including the operating frequency and sensitivity are determined by their architecture. The latter is normally adjusted by trial and error in terms of combining a few basic components such as circular or square resonators, stubs, slots, etc. This is accompanied by parametric analysis guided by engineering experience. At the same time, the design process is most often oriented towards implementing operating parameters (target frequency and/or increasing the resonance depth for resonator-based sensors^28^). Direct enhancement of the device’s sensitivity is rare^29^. Notwithstanding, hands-on sensor development considerably limits the number of potential architectures that can be inspected. Also, these architectures—due to being based on a small set of underlying components—often closely resemble each other. More versatile techniques include topology optimization, where the space assigned to a device is discretized, e.g., split into pixels, which may be associated with metallization of left empty^30–33^. Another option includes pixel devices (e.g., antennas), in which the system architecture is determined by optimizing connections between pre-defined metallic cells^34–36^. The free-form topology optimization offers enhanced flexibility in terms of realizing almost arbitrary parameterization of the structure metallization^37–40^. Many of these techniques utilize custom electromagnetic solvers to improve computational efficiency^41^. These frameworks employ artificial intelligence (AI) tools such as nature-inspired algorithms and/or machine learning procedures. Yet, they have been mostly developed for designing antenna systems. An example can be found in^42^, where an evolutionary algorithm combined with gradient-based search routines was utilized to carry out unsupervised design of compact antennas (multi-band, broadband, etc.) using elementary building blocks in the form of movable rectangular patches and holes of adjustable sizes. Development of AI-based tools for the design of microwave sensors is highly desirable and may lead to the creation of high-performance structures, the properties of which surpass those developed using conventional techniques.

This study introduces an innovative methodology for the unsupervised design of resonator-based microwave sensors realized in microstrip technology. According to our methodology, the geometry of the sensor under design evolves by accommodating a variety of potential building blocks, such as circular and square resonators, stubs, and slots, all being of adjustable size and spatial allocation. The sensor’s topology is determined through Boolean operations over these building blocks. The design process is governed by artificial intelligence (AI) tools, specifically evolutionary algorithms, as well as conventional numerical optimization methods (specifically, gradient-based constrained optimizers). The former controls the topological arrangement of the sensor, whereas the latter allows for explicit enhancement of the sensor’s sensitivity. The design process is driven by performance specifications, with the user determining the target operating frequency of the device and a set of building blocks to be utilized. The design process is unsupervised once initiated, it does not require any designer’s interaction. The presented technique has been demonstrated through the design of a family of high-performance sensors, some of which were experimentally validated and calibrated to corroborate their utility in terms of material characterization. Furthermore, the superiority of AI-generated sensors over state-of-the-art designs reported in the literature is showcased through extensive benchmarking.

The key contributions of this work include:

1. This study addresses the challenge of designing high-performance microstrip sensors for the characterization of dielectric materials without manual intervention. Traditional techniques^13–27^ often require manual tuning and expertise, which can be time-consuming and may not always yield optimal results.
2. To automate the design process, the proposed method uses AI-driven tools, particularly evolutionary algorithms and gradient-based optimizers for final tuning.
3. One of the critical and original components of the proposed design strategy is parameterization of the sensor, which includes a carefully selected set of fundamental cells. This allows for a huge variety of possible circuit architectures while maintaining simplicity in terms of a limited number of adjustable parameters. Consequently, the proposed approach can be categorized as a knowledge-based one.
4. The design approach is exclusively guided by specifications to improve sensor sensitivity and explicitly achieve desired operating frequencies. This strategy, which focuses on objectives, ensures that the sensors produced meet performance requirements without any operator intervention.
5. The research showcases substantial improvements in sensitivity augmentation and frequency optimization compared to the most advanced designs documented in the literature^27,50–54^, achieved through rigorous validation, calibration, and experimentation.
6. The paper indicates several areas for future investigation, such as improving AI-based design methodologies, broadening the range of operating frequencies, studying scalability and applicability to different sensor types and applications, and incorporating advanced machine learning techniques like deep learning to enhance design and optimization.

## Resonator-based microwave sensors

**Fig. 1:**
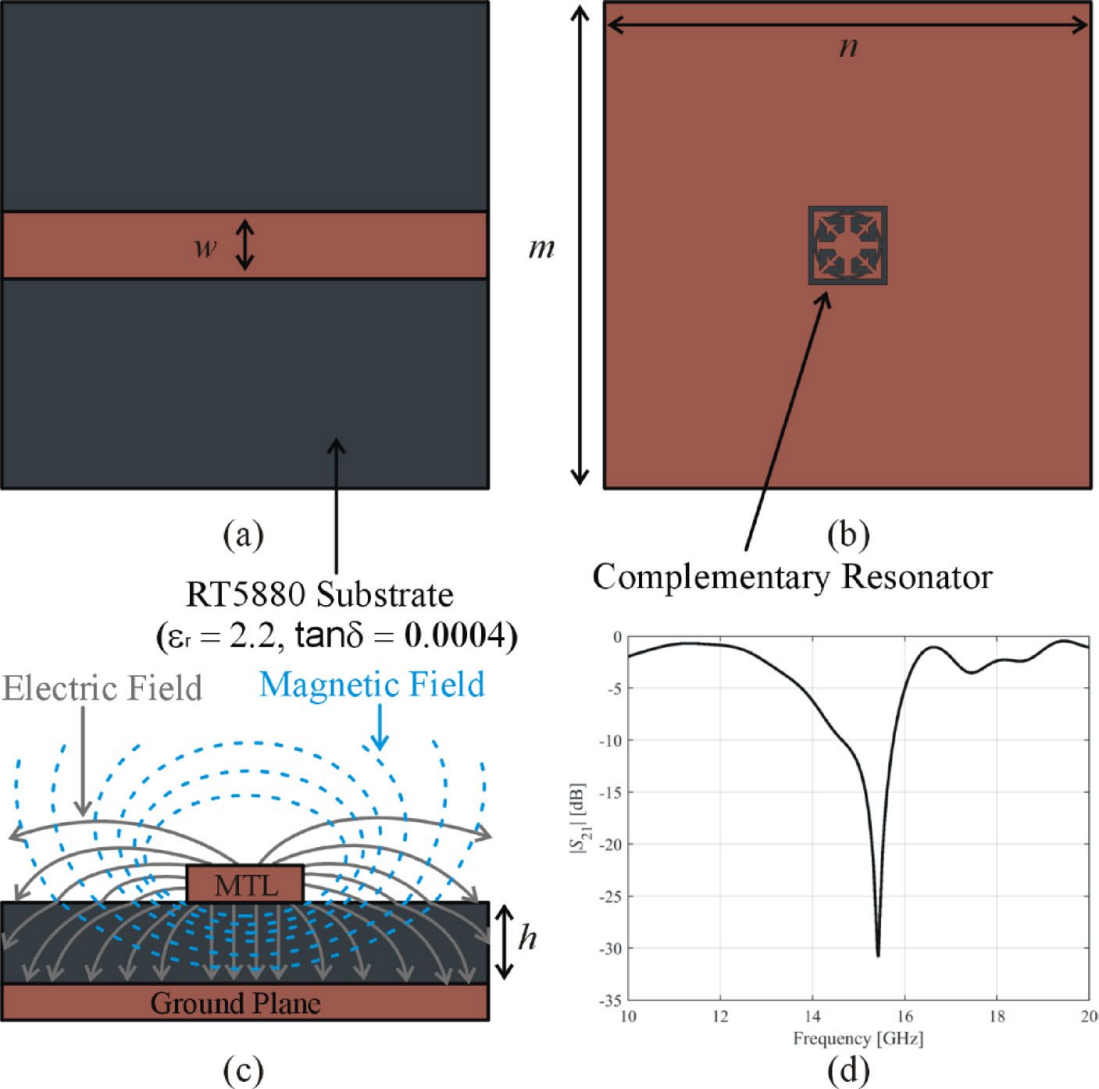
Microstrip sensors: (**a**) microstrip transmission line (MTL) imprinted on the RT5880 substrate’s upper layer, (**b**) complementary resonator etched in the ground plane, (**c**) orientation of the electromagnetic fields produced by the MTL, (**d**) transmission response of the complementary resonator-based microwave sensor.

Microwave sensors based on complementary resonators detect changes in physical parameters, such as temperature, pressure, humidity, or proximity of objects, by measuring variations in the dielectric characteristics, impedance, or electromagnetic field distribution within resonators. Typically, these sensors are constructed on a dielectric substrate that has a conductive microstrip transmission line (MTL) printed on one side and a complementary resonator on the other side, as shown in Fig. 1.

The design process for planar microwave sensors requires meticulous attention to the selection of substrate material, size, impedance matching, and the architecture of complementary resonators^43^. The substrate employed in this study is RT5880 (*ε*_*r*_ = 2.2 ± 0.02, tan*δ* = 0.0004) material suitable for high-frequency applications.

The arrangement of complementary resonators beneath the 50 Ω MTL provides for an efficient coupling between the resonators and the electromagnetic waves produced by the MTL, as shown in Fig. 1(c). This coupling leads to a narrow stopband with a strong rejection, as shown in Fig. 1(d). The stopband properties of these resonators can be employed to detect changes in the surrounding environment. The main design goals for microwave sensors are high sensitivity, linearity, stability, and reproducibility in measurements. Designers iteratively modify parameters such as substrate material, resonator dimensions, and geometry to get the desired performance.

As mentioned earlier, the development of microstrip sensors is a tedious process that requires a careful selection of the circuit geometry and its dimensions, which is normally carried out through trial and error. Furthermore, it is oriented towards achieving a specific operating frequency (and, optionally, satisfactory resonance depth), none of which can directly control the most important performance parameters, which is sensitivity. The unsupervised design methodology introduced in Section “Unsupervised sensor design: methodology” aims at alleviating these difficulties and enabling the automated development of high-performance sensors without any interaction with the human expert.

## Unsupervised sensor design: methodology

This section presents our proposed method for the unsupervised design of resonator-based microstrip sensors. We begin by outlining the fundamental assumptions in Section “Basic assumptions”. Sensor parameterization is discussed in Section “Sensor parameterization”, followed by an elucidation of the computational model in Section “EM model”. Sections “AI-based sensor evolution” and “Final parameter adjustment” delve into the evolution of sensor geometry and dimensions through AI techniques, as well as fine-tuning for sensitivity enhancement. Finally, the overall operation of the algorithm is summarized in Section “Complete algorithm”.

### Basic assumptions

The AI-based design methodology introduced in this study is developed using the following set of fundamental assumptions. To begin with, the sensor is implemented on a specific substrate, which is kept fixed during the design process. Here, we use low-loss 0.51-mm-thick RT5880 material featuring relative permittivity of *ε*_*r*_ = 2.2. The overall structure follows the guidelines discussed in Section “Resonator-based microwave sensors”, in particular, we have a microstrip line on the back side of the device, whereas the resonators are etched on the front layer. The sensor’s geometry is determined by allocating elementary building blocks (to be introduced in Section “Sensor parameterization”), which are of adjustable size, and can be enabled (i.e., present) or disabled (removed from the structure). The allocation and sizing of these unit cells are governed by the evolutionary algorithm discussed in Section “AI-based sensor evolution”. The sensor evolution process will be followed by local tuning (arranged using a gradient-based procedure), the aim of which is explicit improvement of the sensor’s sensitivity. The computational models involved in the process are explained in Section “EM model”.

### Sensor parameterization

The design methodology proposed in this study assumes that the microstrip sensor under development incorporates a certain number of basic components, which include complementary square and circular resonators, stubs, and slots. These components undergo size modifications and integration to form complex geometries. It is also possible to disable particular unit cells by assigning them zero sizes. Table 1 provides the geometric parameters of the aforementioned building elements.

**Fig. 2:**
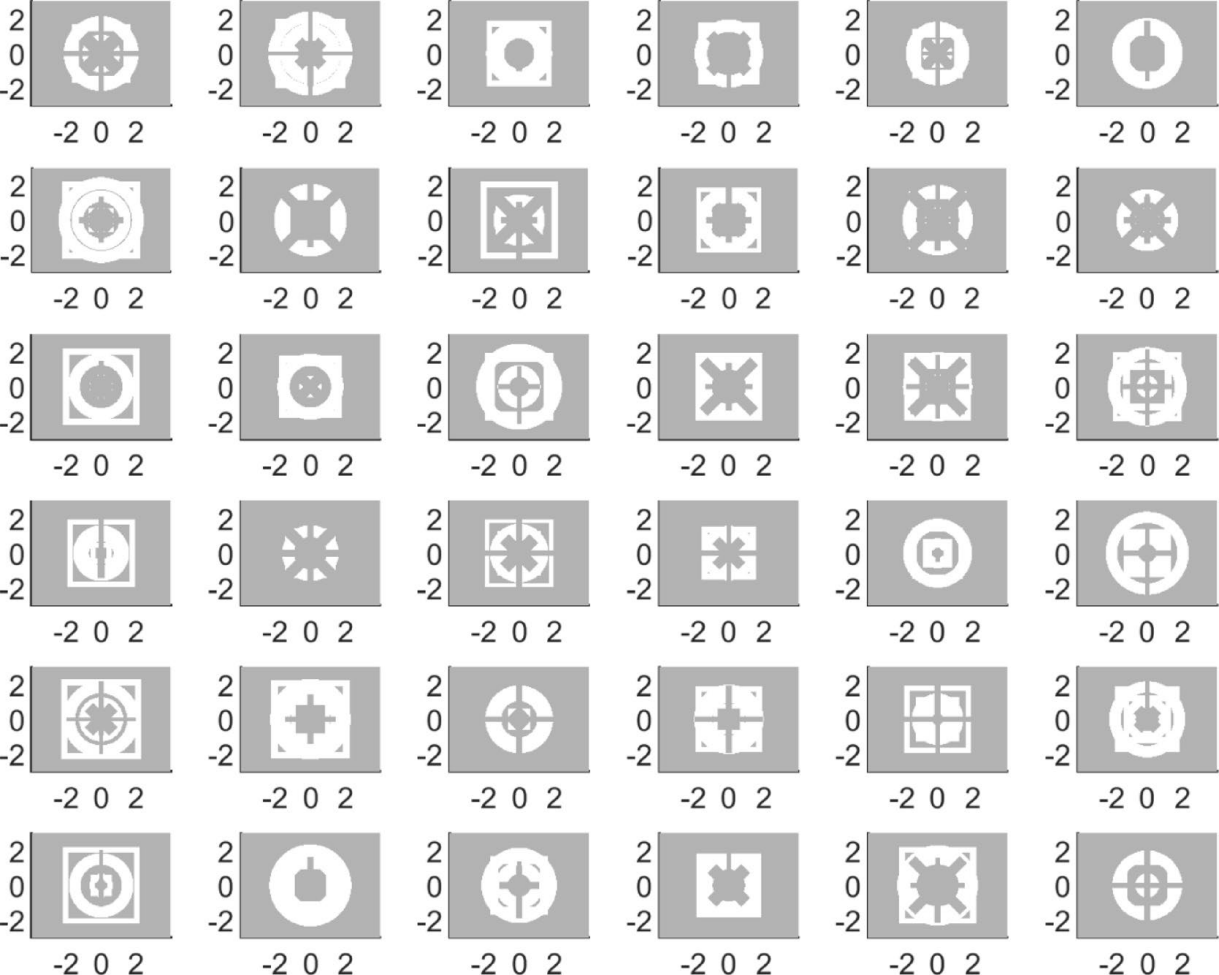
A selection of randomly generated sensor geometries utilizing the components enlisted in Table 1. These geometries indicate the ability to generate a large variety of complex geometries by utilizing a few essential building elements. The sizes as marked on the picture axes are in millimeters.

As illustrated in Fig. 2, the considered elementary building elements are sufficient to create essentially an infinite number of diverse sensor topologies of various shapes, which is indicative of the possibility of developing high-performance devices through appropriate evolution and optimization. Both will be elucidated in detail in Sections “EM model” through “Complete algorithm”.

There are two main reasons for selecting the proposed sensor parameterization approach. The first reason is associated with computational efficiency: sensor’s evolution requires solving computationally heavy optimization tasks where all decision variables are simultaneously adjusted. The number of parameters determines the complexity of the problem and the CPU time required to solve it. Alternative representations, such as pixel-based structures, feature excessive amounts of decision variables, and to achieve sufficient resolution of the device’s geometry, the pixel sizes must be small enough, and, consequently, their number must be large. On the other hand, the proposed technique uses a small number of parameters (only sixteen), yet enables a huge number of potential architectures, as illustrated in Fig. 2. This translates into computational efficiency, as the evolution process only requires about 1000 EM simulations. The second reason is flexibility. Compared to other options (e.g., pixel-based structures), our technique uses continuous parameters, which enable a large variety of possible topologies but also allow for fine tuning using local algorithms. Furthermore, the specific selection of building blocks (e.g., square and circular resonators) incorporates problem-specific knowledge, which reduces computational complexity without compromising the number of possible options to be considered.

**Table 1:** Geometric parameters of the basic Building elements of the microstrip sensor.

Component name	Geometry	Design variables	Lower variable bounds [mm]	Upper variable bounds [mm]
Square 1		*as*_11_ *as*_12_	1.00 0.10	2.00 0.30
Square 2		*as*_21_ *as*_22_	0.01 0.10	1.50 0.30
Circle 1		*ac*_11_ *ac*_12_	1.00 0.20	2.00 0.30
Circle 2		*ac*_21_ *ac*_22_	0.01 0.20	1.50 0.30
Cross 1		*cl*_1_ *cw*_1_	0.1 0.1	2.5 0.3
Cross 2		*cl*_2_ *cw*_2_	0.1 0.1	2.5 0.3
Stub 1		*sl*_1_ *sw*_1_	0.1 0.1	2.5 0.3
Stub 2		*sl*_2_ *sw*_2_	0.1 0.1	2.5 0.3

### EM model

**Fig. 3:**
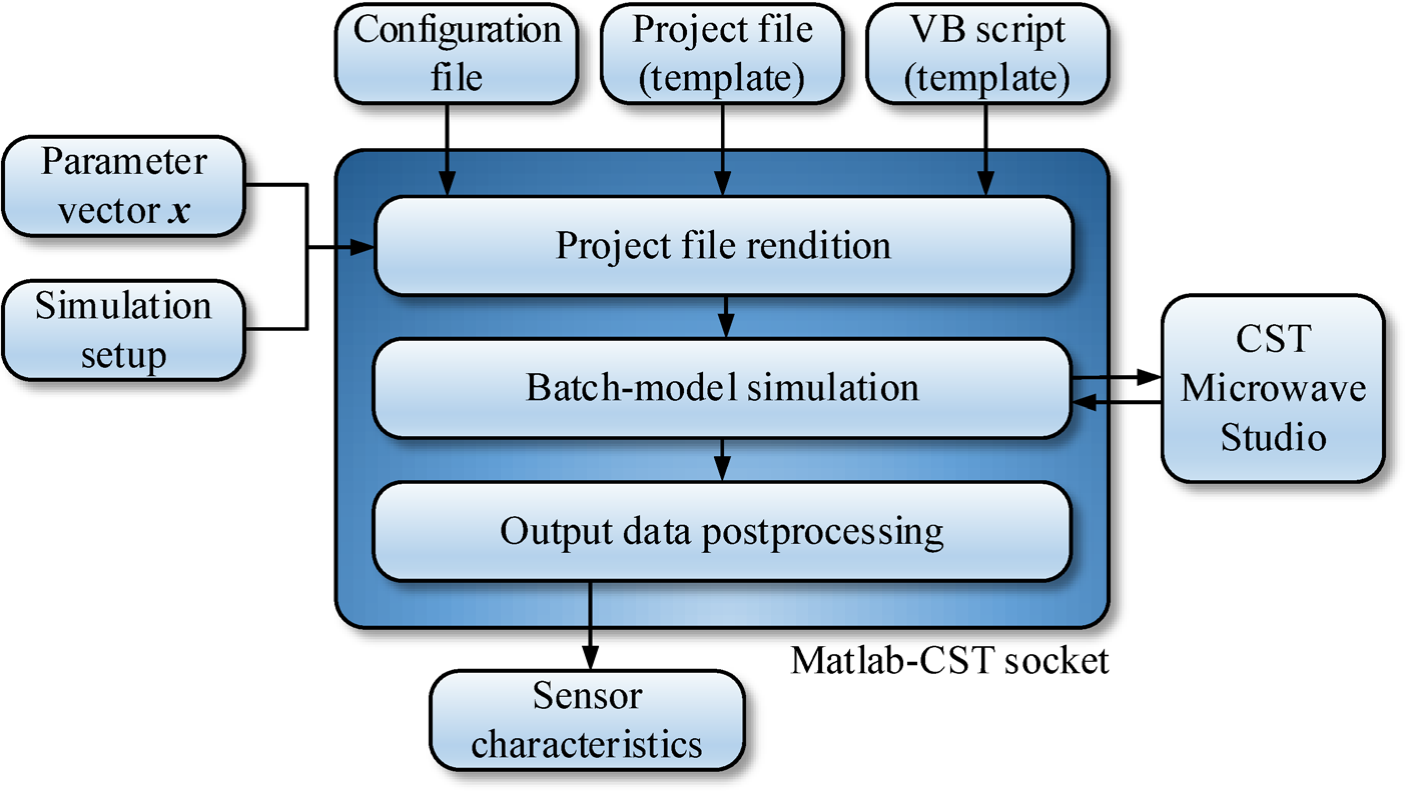
Matlab-CST interface employed in this study. Using the sensor’s geometry parameters and data pertinent to EM analysis setup, a working CST file is generated based on the raw (template) .cst project and the template Visual Basis script. The EM analysis is carried out, and the data post-processing is realized to prepare the final results of sensor evaluation (in particular, its transmission response).

**Fig. 4:**
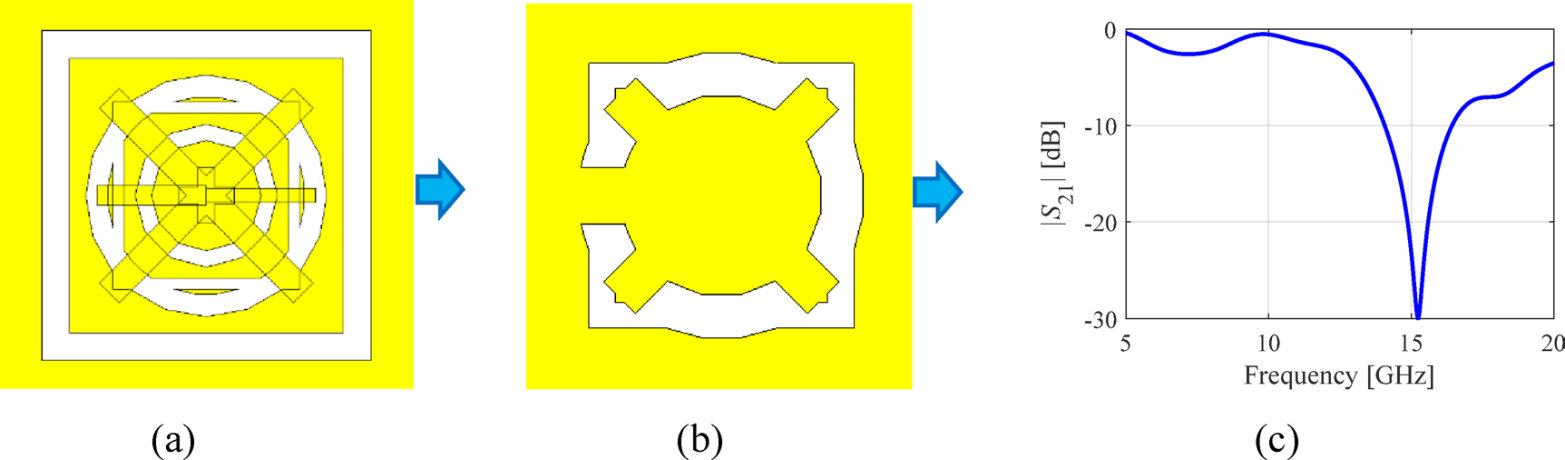
Evaluating simulation model (here, realized and simulated in CST Microwave Studio): (**a**) raw model showing all elementary building blocks, (**b**) specific sensor structure rendered for a particular parameter vector ***x***, (**c**) transmission response obtained through EM simulation.

The underlying computational model utilized in this study is CST Microwave Studio^43^. The raw EM model implements all building blocks of the sensor as discussed in Section “Sensor parameterization”. As demonstrated, this set of unit cells allows us to generate a large variety of sensor architectures, the complexity of which can be controlled by enabling/disabling particular components. In practice, to disable a component, it is assigned a zero size. While evaluating the sensor frequency characteristics, the vector of parameters ***x*** is assigned to adjust the sizes of individual components and to realize the specific topology represented by ***x***. EM simulation is performed in a batch mode using a custom-designed socket between Matlab^44^ and CST Microwave Studio, outlined in Fig. 3. The key component of the interface is a Visual Basic script generated on the fly to set the value of the sensor parameters according to the evaluation vector ***x***. Other components are utilized to run the simulation and extract the results. The simulation process entails rebuilding the original (raw) computational model to represent a sensor’s topology corresponding to the vector ***x***, EM analysis, and post-processing the output data, as indicated in Fig. 4.

**Fig. 5:**
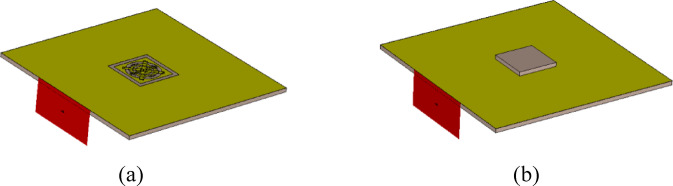
Computational model: (**a**) unloaded sensor ***R***_*EM.*0_(***x***), (**b**) sensor loaded with a dielectric sample of the size 5 × 5 × 0.51 mm (*ε*_*r*_ = 2.2), denoted as ***R***_*EM.*1_(***x***).

In the design process, two EM models are employed, one corresponding to the unloaded sensor, denoted as ***R***_*EM*.0_(***x***), and another one, corresponding to the sensor loaded with a dielectric sample of the size 5 × 5 × 0.51 mm (relative permittivity *ε*_*r*_ = 2.2), denoted as ***R***_*EM*.0_(***x***), cf. Figure 5. Evaluation results of both models allow us to directly compute the sensor’s sensitivity using the formula

1 $$S({\mathbf{x}})=\frac{{{f_r}({{\mathbf{R}}_{EM.0}}({\mathbf{x}})) - {f_r}({{\mathbf{R}}_{EM.1}}({\mathbf{x}}))}}{{{f_r}({{\mathbf{R}}_{EM.0}}({\mathbf{x}}))\left[ {{\varepsilon _r} - 1} \right]}}\;$$

where *f*_*r*_(***R***_*EM.j*_(***x***)) is the resonant frequency of the unloaded and loaded sensor (*j* = 0 and 1, respectively).

### AI-based sensor evolution

**Fig. 6:**
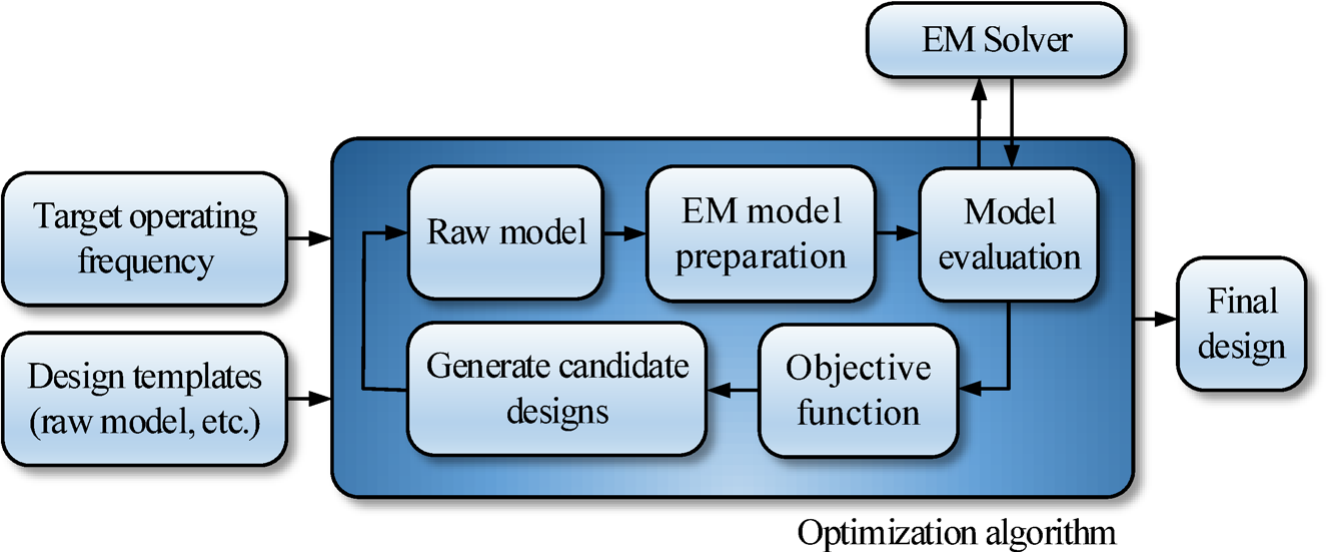
Generic flow diagram of unsupervised microstrip sensor development by means of optimization tools and parameterization of Sections “Sensor parameterization” and “EM model”.

The unsupervised microstrip sensor design process is realized in two stages. In the first stage, a global search procedure is launched, which aims at simultaneous adjustment of the sensor architecture and dimensions. Leveraging the properties of sensor parameterization introduced in Sect. 2.2, this boils down to a continuous optimization of the parameter vector ***x***. The second stage is local parameter tuning oriented towards explicit enhancement of the sensor’s sensitivity (cf. Section “Final parameter adjustment”). The operating flow of the sensor’s development process has been shown in Fig. 6. Note that the diagram represents both stages. The only difference is the specific optimization algorithm. Note that the process is entirely specification driven (here, represented as the target operating frequency). All supplementary data (dielectric substrate, geometric details of the complete device and the microstrip line, etc.) are encoded in the raw EM model supplied by the user.

#### Design task

The design problem to be addressed during the first stage is formulated as follows

2 $${{\mathbf{x}}^*}=\arg \mathop {\hbox{min} }\limits_{{{\mathbf{x}} \in X}} {U_{global}}({\mathbf{x}})$$

In (4), x^*^ is the optimum design to be found. The design variable space *X* is defined using the sensor variable bounds. The analytical form of the merit function *U*_*global*_ is

3 $${U_{global}}({\mathbf{x}})=\left\{ \begin{gathered} |{S_{21}}({\mathbf{x}},{f_t})|\;\;{\text{if}}\;\;|{S_{21}}({\mathbf{x}},{f_t})|> - 20{\text{dB}} \hfill \\ |{S_{21}}({\mathbf{x}},{f_t})| \cdot \left[ {1+S({\mathbf{x}})} \right]\;\;\;{\text{otherwise}} \hfill \\ \end{gathered} \right.$$

In (3), *f*_*t*_ is the target operating frequency. The transmission response *S*_21_ is evaluated using the unloaded sensor (model ***R***_*EM.*0_(***x***)), whereas sensitivity *S*(***x***) is obtained as in (1). Note that the second part of *U*_*global*_ requires evaluation of both unloaded and loaded sensor models, but it is only carried out if the transmission response at the target frequency *f*_*t*_ falls below − 20 dB.

It should be reiterated that the sensor parameterization of Sect. 2.2 has a fundamental advantage of the vector ***x*** simultaneously determining the circuit’s architecture and dimensions. This allows us to concurrently adjust both in a single global optimization process, and then execute fine tuning using local algorithms (cf. Section “Final parameter adjustment”).

#### Evolutionary algorithm

**Fig. 7:**
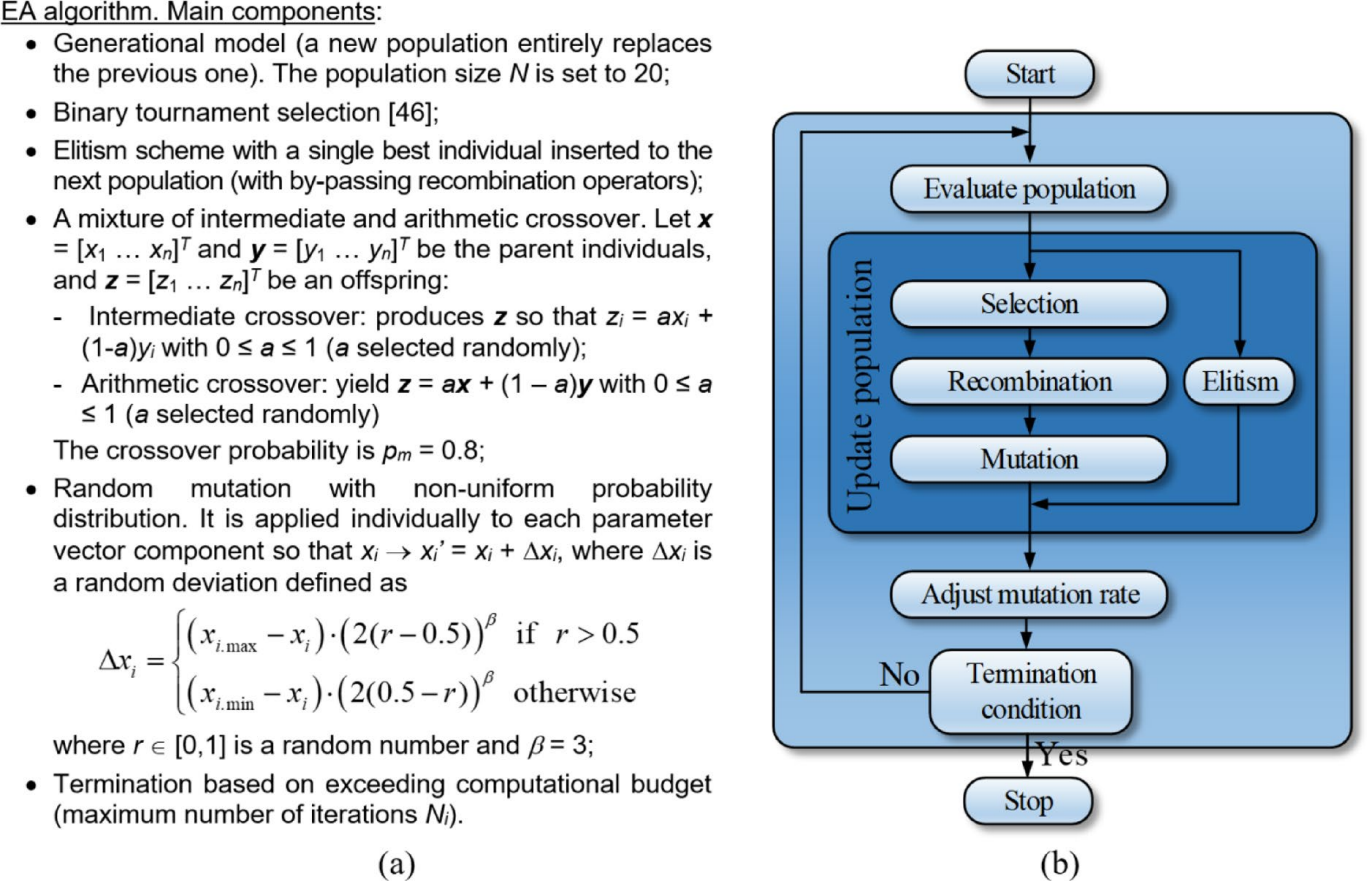
Evolutionary algorithm (EA) utilized for unsupervised design of microstrip sensors using parameterization of Section “Sensor parameterization” and computational model of Section “EM model”: (**a**) main algorithm components, (**b**) flow diagram.

**Fig. 8:**
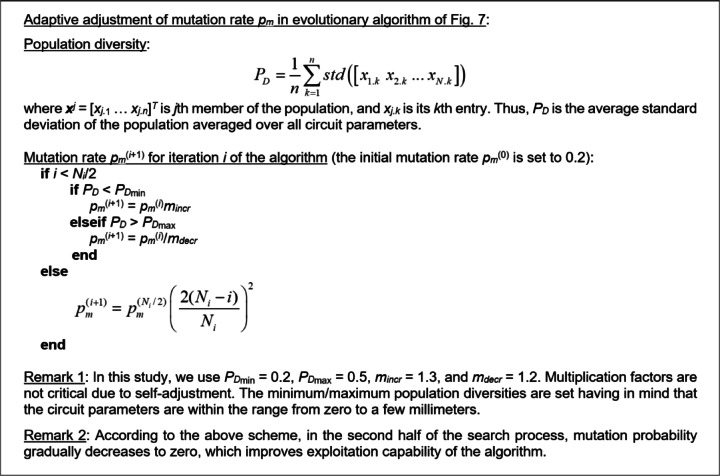
Adjusting mutation probability in the EA of Fig. 7.

The sensor evolution and global optimization stage is realized using a floating-point evolutionary algorithm (EA)^45,46^ outlined in Fig. 7. The algorithm employs elitism and adaptive adjustment of the mutation rate to facilitate precise optimum identification at the later stages of the search process (cf. Figure 8). In particular, gradually reducing the mutation rate at late stages of the search process suppresses the number of random changes of the candidate designs, thereby promoting exploitation of the design variable space region identified in the process. At the same time, larger changes of the sensors topology are limited as well. This translates into additional improvements of the objective function, as opposed to reaching a plateau towards the end of the search run (see also Fig. 11).

### Final parameter adjustment

In the second design stage we explicitly enhance the sensor’s sensitivity while maintaining the target center frequency *f*_*t*_ (of the unloaded sensor). The objective function handled at this stage is *U*_*local*_ defined as

4 $${U_{local}}({\mathbf{x}})= - S({\mathbf{x}})+\beta {\left[ {{f_r}({{\mathbf{R}}_{EM.0}}({\mathbf{x}})) - {f_t}} \right]^2}$$

where the sensitivity *S*(***x***) has been defined in (3), whereas *β* is a penalty coefficient, here, set to 10. As indicated in (4), the main goal is improvement of sensitivity, whereas the second (penalty) term is introduced to enforce the (unloaded) operating frequency to stay at *f*_*t*_.

**Fig. 9:**
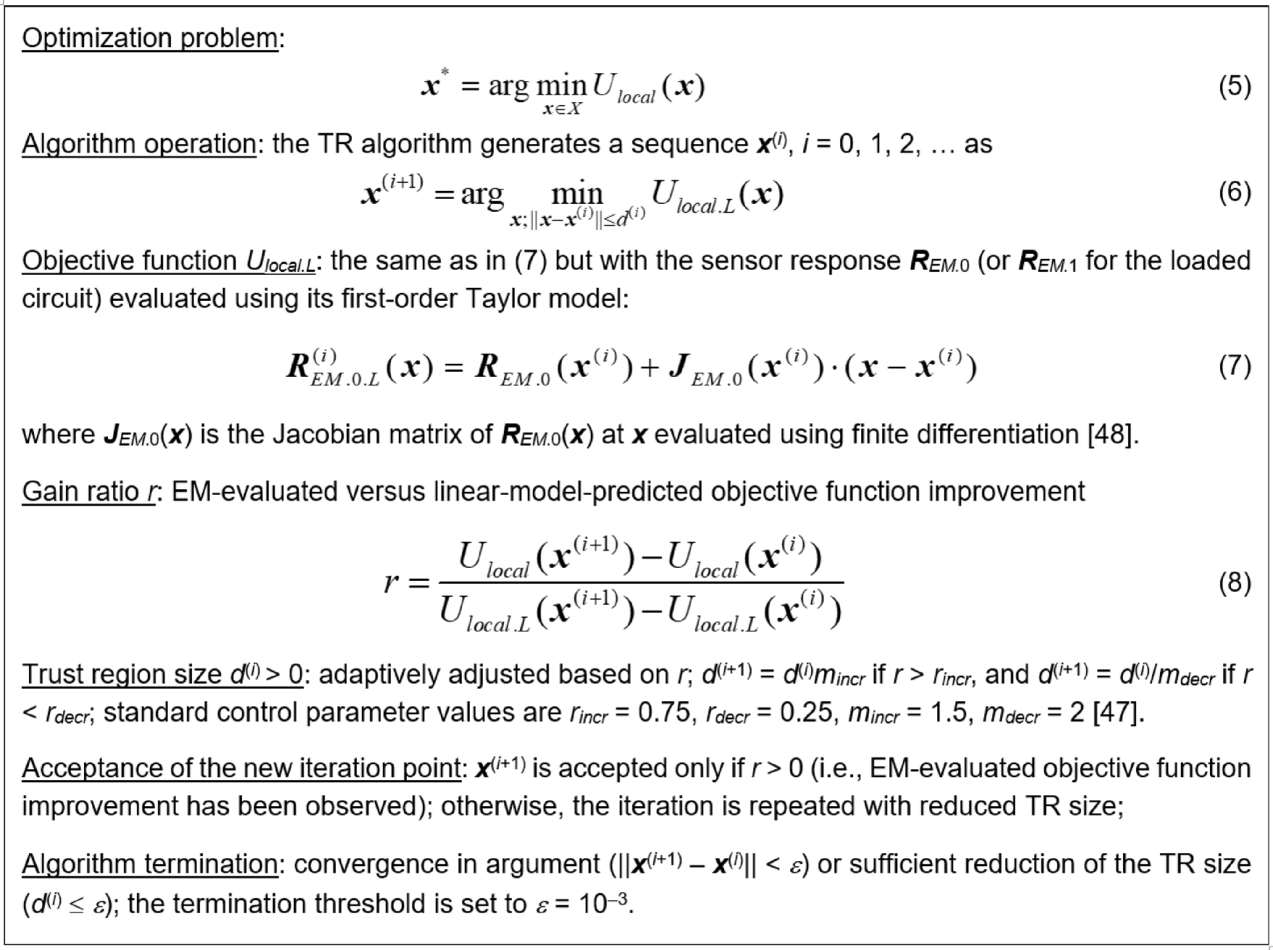
The outline of the TR algorithm for final adjustment of sensor’s geometry parameters.

Here, local optimization is executed using is a trust-region (TR) gradient-based algorithm^47^ with numerical derivatives^48^, outlined in Fig. 9. The initial design ***x***^(0)^ is the one produced by the evolutionary algorithm. The TR algorithm yields a sequence ***x***^(*i*)^, *i* = 0, 1, …, of parameter vectors approximating the optimum x^*^ using a first-order Taylor expansion model of the sensor’s outputs constructed at ***x***^(*i*)^. The local optimization task (6) is resolved by means of a Sequential Quadratic Approximation (SQP) algorithm^49^ (here, we use implementation available in the Matlab Optimization Toolbox^44^).

Again, it should be emphasized that owing to the parameterization introduced in Sect. 2.2, the same parameter vector ***x*** is utilized in both global and local search stages. On the one hand, large-scale alterations of the sensor’s parameters mainly affect the circuit architecture. On the other hand, local changes (as in gradient-based optimization) allow us to control the devices responses without changing its overall topology.

**Fig. 10:**
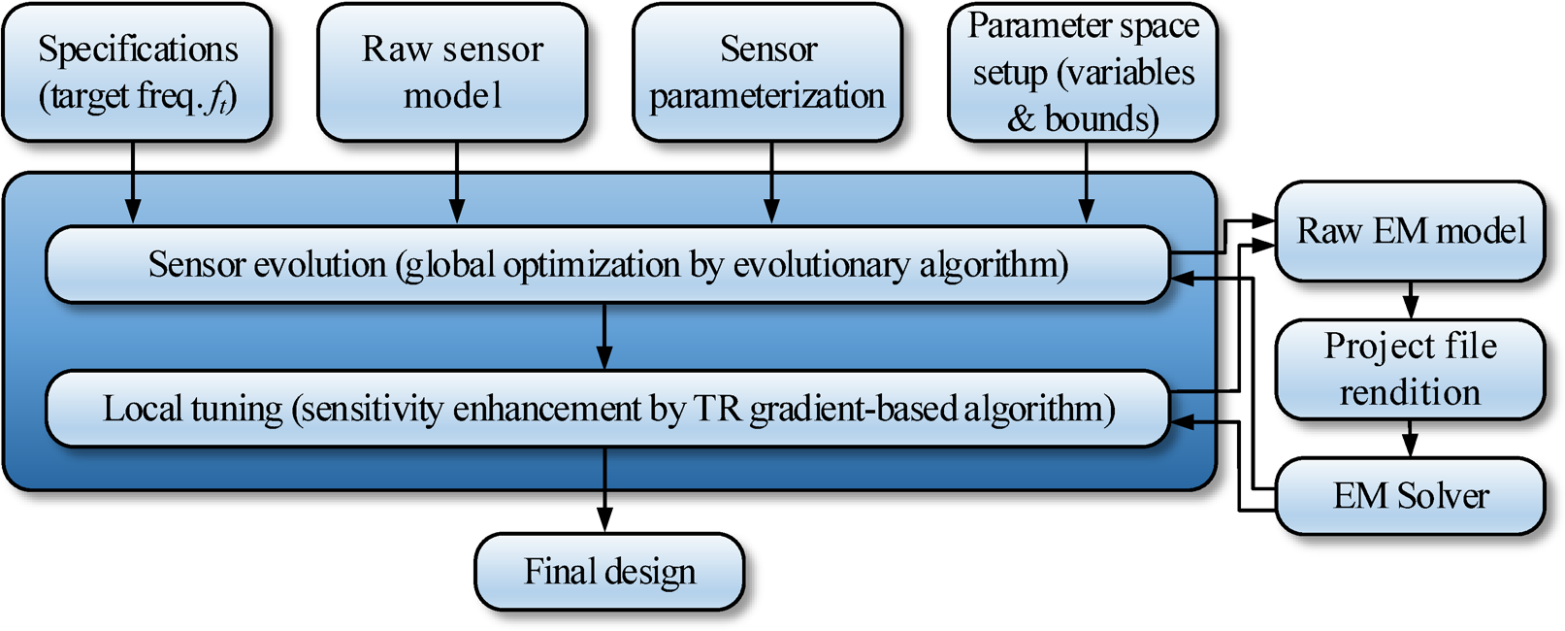
Unsupervised microstrip sensor design: flow diagram.

### Complete algorithm

The complete algorithm for unsupervised design of microstrip sensors proposed in this study has been shown in Fig. 10. The process is automated and only necessitates—as its inputs—the target operating frequency, and the raw computational model, which encapsulates components such as the microstrip line implemented on the substrate of choice, as well as building blocks of the circuit as elaborated on in Section “Sensor parameterization”.

The specific sensor topology and the circuit’s dimensions are adjusted through two consecutive stages, a global search (architecture evolution) and local tuning (final adjustment of circuit’s geometry parameters). Both stages are driven by their corresponding objective function, which are defined (cf. (3) and (4)) to promote designs that exhibit target operating frequency with deep notch depth and maximize the sensor’s sensitivity.

**Fig. 11:**
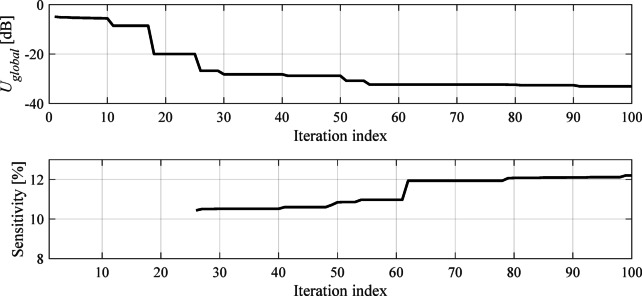
Typical evolution of the objective function *U*_*global*_ (3) at the global optimization stage. Top: objective function, bottom: evolution of circuit sensitivity. Recall that sensitivity is being evaluated only for designs with sufficiently good transmission response.

**Fig. 12:**
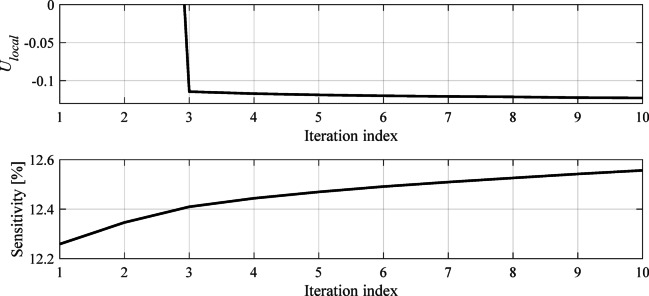
Typical evolution of the objective function *U*_*local*_ (4) at the final tuning stage. The local optimization stage normally takes a few iterations (ten in this example). The sensitivity is further improved, whereas the required center frequency is maintained by means of the penalty factor (cf. (4)).

It should be noted that dividing the optimization process into two phases (global and local) allows for more efficient operation of the framework. In the first phase, the primary objective is sensor architecture evolution as well as bringing its operating frequency to the assumed target. In the second stage, the circuit’s sensitivity is explicitly optimized while maintaining its operating frequency. At this stage, large-scale adjustments of the circuit topology are no longer occurring. Figures 11 and 12 show typical evolution of the global and local objective function, as well as the improvement of the circuit sensitivity in the course of the optimization process.

It should also be emphasized that the proposed design framework works irrespective of the assumed operating frequency. The frequency is only used in formulating the objective function, meaning that the sensor’s evolution and optimization work regardless of its particular choice. This is evident when looking at the formulation (3) of the global objective function (where the target frequency *f*_*t*_ is a parameter used to evaluate the design quality), as well as the local objective function (4), where, again, *f*_*t*_ is used for the same purpose. This means that the center frequency selected for the purpose of illustration in Section “Demonstration examples” could have been changed to any value the user wished the sensor to be designed for. To lower the operating frequency, one also needs to adjust the bounds of the design variables (cf. Table 1) to make the structure physically realizable. For example, the upper bounds should be enlarged for lower values of the target operating frequency. At the same time, it should be emphasized that the material parameters of the substrate are also just design parameters, which can be changed according to the designer’s needs.

A comment should be made concerning the computational cost of the sensor design process. The cost of the first part (topology evolution) is 1200 EM simulations (60 iterations of the evolutionary algorithm with the population size of 20), whereas local tuning typically takes up to 200 EM analyses (instance dependent). The evaluation time of the EM model is typically 40 s at the global search (where coarser-discretization is employed), and about one minute at the fine-tuning stage (using fine-discretization analysis). Thus, the total cost is around 16 h, which is practically acceptable given the fact that the process is entirely unsupervised.

## Demonstration examples

For demonstration purposes, the unsupervised design framework introduced in Section “Unsupervised sensor design: methodology” has been employed to develop sensors operating at various frequencies (3 GHz, 6 GHz, 9 GHz, 12 GHz, and 15 GHz) and a few high-performance microstrip sensors. Selected examples are briefly outlined in this section. In all cases, the evolutionary algorithm (global search stage) and the local tuning procedure have been executed using the setup detailed in Section “Unsupervised sensor design: methodology”.

**Fig. 13:**
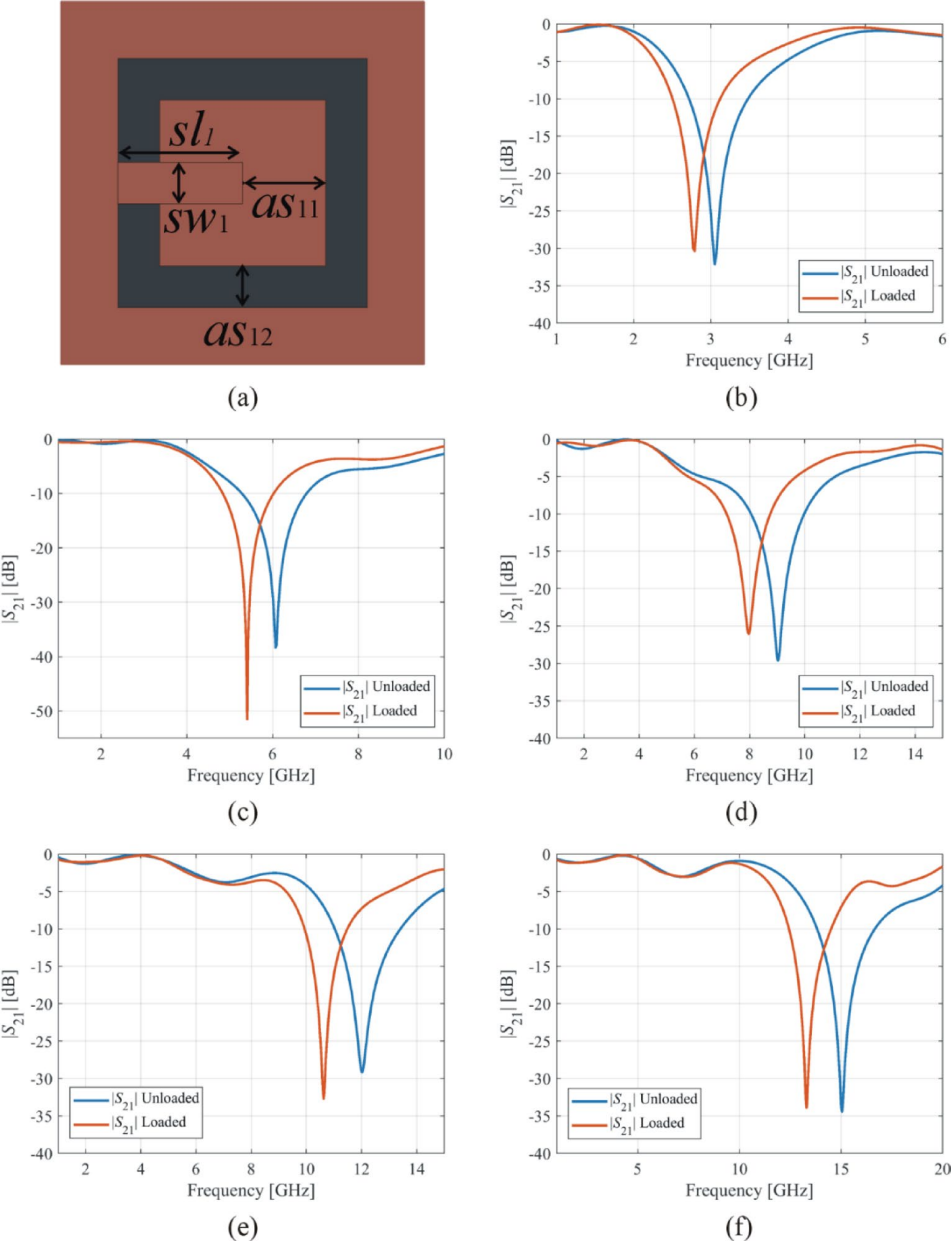
(**a**) Resonator geometry for 3 GHz, 6 GHz, 9 GHz, 12 GHz, and 15 GHz sensors, (**b**) transmission coefficients (*S*_21_) for unloaded and loaded 3 GHz sensor, (**c**) *S*_21_ for unloaded and loaded 6 GHz sensor, (**d**) *S*_21_ for unloaded and loaded 9 GHz sensor, (**e**) *S*_21_ for unloaded and loaded 12 GHz sensor, and (**f**) *S*_21_ for unloaded and loaded 15 GHz sensor.

The simple resonator based on a square ring (as11, as12) and a stub (sl1, sw1) has been used to design optimized sensors operating at various frequencies, as shown in Fig. 13(a). By employing these four geometric parameters (*as*_11_, *as*_12_, *sl*_1_, and *sw*_1_) and the evolutionary algorithm, the geometric parameters for 3 GHz, 6 GHz, 9 GHz, 12 GHz, and 15 GHz sensors are obtained and summarized in Table 2. To determine the relative sensitivity of these sensors operating at different frequencies, each sensor’s resonator is loaded with the RO5880 substrate as a material under test (MUT). The dimensions of the MUT are 7.5 × 7.5 × 0.51 mm. The transmission coefficients of the sensors, both unloaded and loaded with the MUT, are displayed in Fig. 13(b), (c), (d), (e), and (f) for frequencies of 3 GHz, 6 GHz, 9 GHz, 12 GHz, and 15 GHz, respectively. Table 3 compares the resonant frequencies, geometrical sizes, and sensitivity of the sensors operating at 3 GHz, 6 GHz, 9 GHz, 12 GHz, and 15 GHz. As the frequency of operation increases, the relative sensitivity remains generally constant, with values averaging approximately 9–10% for frequencies between 6 GHz and 15 GHz, and slightly lower at 3 GHz (7.12%). The optimized geometric parameters exhibit an inverse correlation with the operating frequency, indicating the requirement for reduced dimensions at higher frequencies. The reduced dimensions of the resonator pose challenges in both the manufacturing process and the accuracy of measurements. Thus, using only one resonator constructed with a square ring and a stub is inadequate for achieving the desired dimensions and a sensitivity above 10%. The subsequent examples employ numerous fundamental resonators listed in Table 1 to generate high-sensitivity complex structures. It should be mentioned that achieving higher sensitivity is facilitated by selecting a higher operating frequency, which also improves the sensor’s predictive power in terms of estimating material parameters due to larger frequency variations between unloaded and loaded devices.

**Fig. 14:**
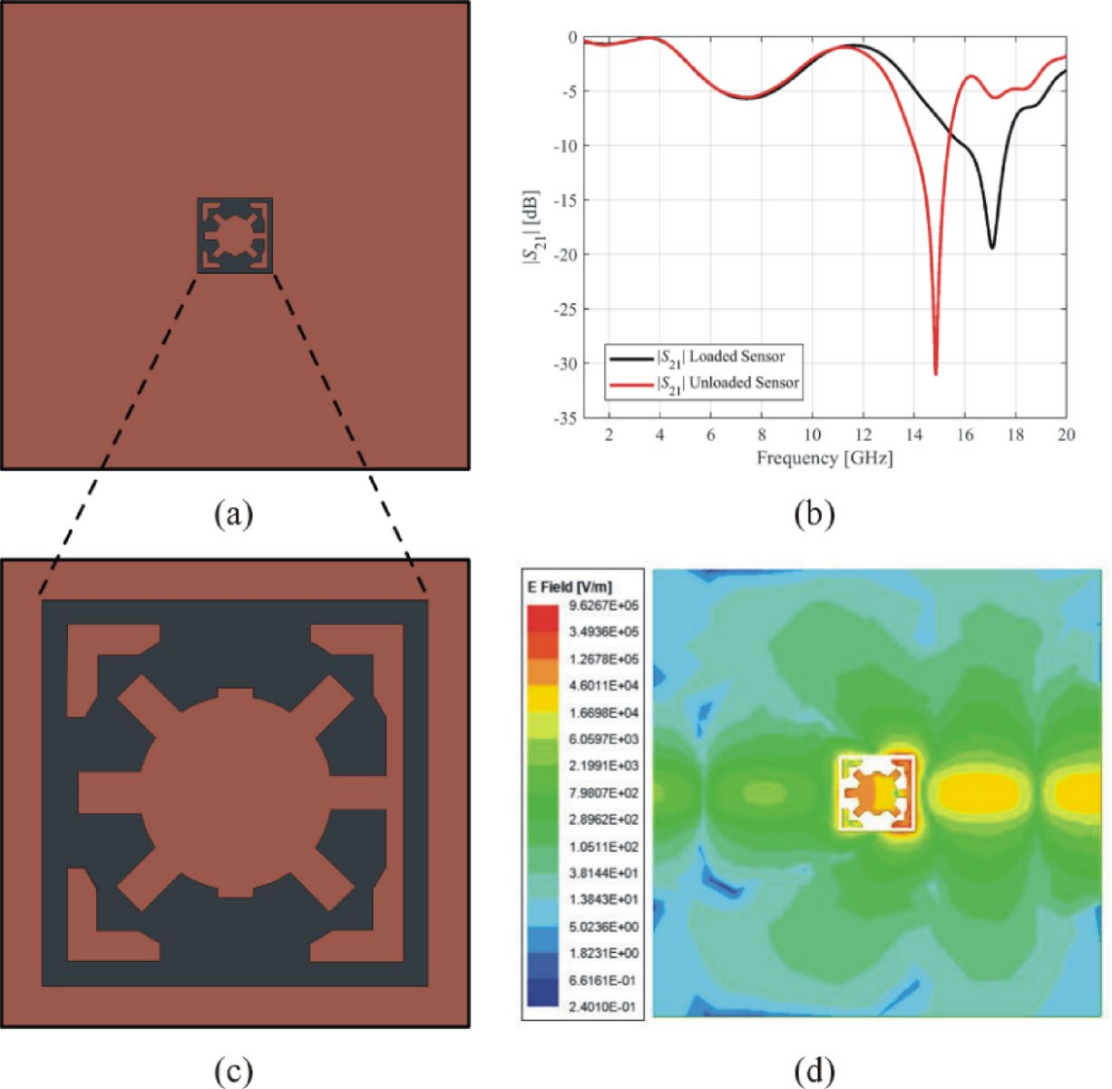
Exemplary Sensor 1 (**a**) ground layer, (**b**) transmission response, (**c**) geometry of the complementary resonator, (**d**) electric field emitted from the complementary resonator at 17 GHz.

The first example of a high-performance microstrip sensor, shown in Fig. 14(a), depicts the ground layer of exemplary Sensor 1. The sensor’s resonant frequency is 17.08 GHz, whereas the notch depth is −19.44 dB, as indicated by the transmission response in Fig. 14(b). The complementary resonator’s geometry for Sensor 1 (cf. Figure 14(c)), whereas the corresponding electric field is illustrated in Fig. 14(d). The field strength is the highest near the edges of the slots and square of Sensor 1, with a maximum magnitude of 9.62 × 10^5^ V/m.

**Table 2:** Geometrical dimensions of the resonator used to design 3 ghz, 6 ghz, 9 ghz, 12 ghz, and 15 ghz sensors.

Geometric variables for sensors	3 GHz sensor (mm)	6 GHz sensor (mm)	9 GHz sensor (mm)	12 GHz sensor (mm)	15 GHz sensor (mm)
*as*_11_	4.95	2.33	1.53	1.11	0.86
*as*_12_	0.5	0.5	0.5	0.5	0.5
*sl*_1_	5.45	2.83	2.03	1.61	1.36
*sw*_1_	0.5	0.5	0.5	0.5	0.5

**Table 3:** Comparison of loaded and unloaded resonant frequencies, geometrical sizes, and sensitivity of 3 ghz, 6 ghz, 9 ghz, 12 ghz, and 15 ghz sensors.

Sensor Designed at Several Frequencies	Unloaded Sensor Resonant Frequency (GHz/dB)	Loaded Sensor Resonant Frequency (GHz/dB)	External Size of Resonator (mm × mm)	Sensitivity (%)
3 GHz sensor	3.04/-32.11	2.78/-30.38	10.90 × 10.90	7.12
6 GHz sensor	6.06/-38.33	5.40/-51.47	5.65 × 5.65	9.07
9 GHz sensor	9.02/-29.61	7.96/-26.05	4.05 × 4.05	9.79
12 GHz sensor	12.02/-29.15	10.65/-32.71	3.21 × 3.21	9.71
15 GHz sensor	15.04/-34.43	13.29/-33.89	2.72 × 2.72	9.70

To determine the sensitivity of Sensor 1, the resonator is loaded with a MUT known as RO5880 substrate (relative permittivity *ε*_*r*_ = 2.2), which has the following dimensions: 5 × 5 × 0.51 mm. The center frequency of the loaded sensor is 14.86 GHz, whereas the notch depth is −31.1 dB. Equation (1) allows for the assessment of sensitivity, which yields a value of 10.82% for Sensor 1.

**Fig. 15:**
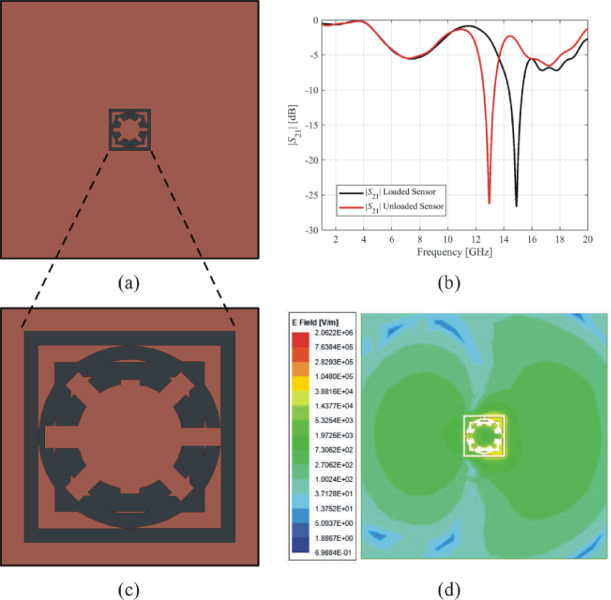
Exemplary Sensor 2 (**a**) ground layer, (**b**) transmission response, (**c**) geometry of the complementary resonator, (**d**) electric field emitted from the complementary resonator at 14.9 GHz.

The ground layer of exemplary Sensor 2 is shown in Fig. 15(a). This sensor’s notch depth is − 26.6 dB, and its resonant frequency is 14.90 GHz, as shown by the transmission response in Fig. 15(b). The geometry of the complementary resonator of Sensor 2 is depicted in Fig. 15(c), and the electric field generated by this sensor is shown in Fig. 15(d). The electric field exhibits its highest intensity near the borders of the inner ring and slots of Sensor 2, reaching a maximum magnitude of 2.06 × 10^6^ V/m. The loaded sensor with the MUT has a resonant frequency of 12.94 GHz with a notch depth of − 26.22 dB. This sensor exhibits a relative sensitivity of 10.95%.

**Fig. 16:**
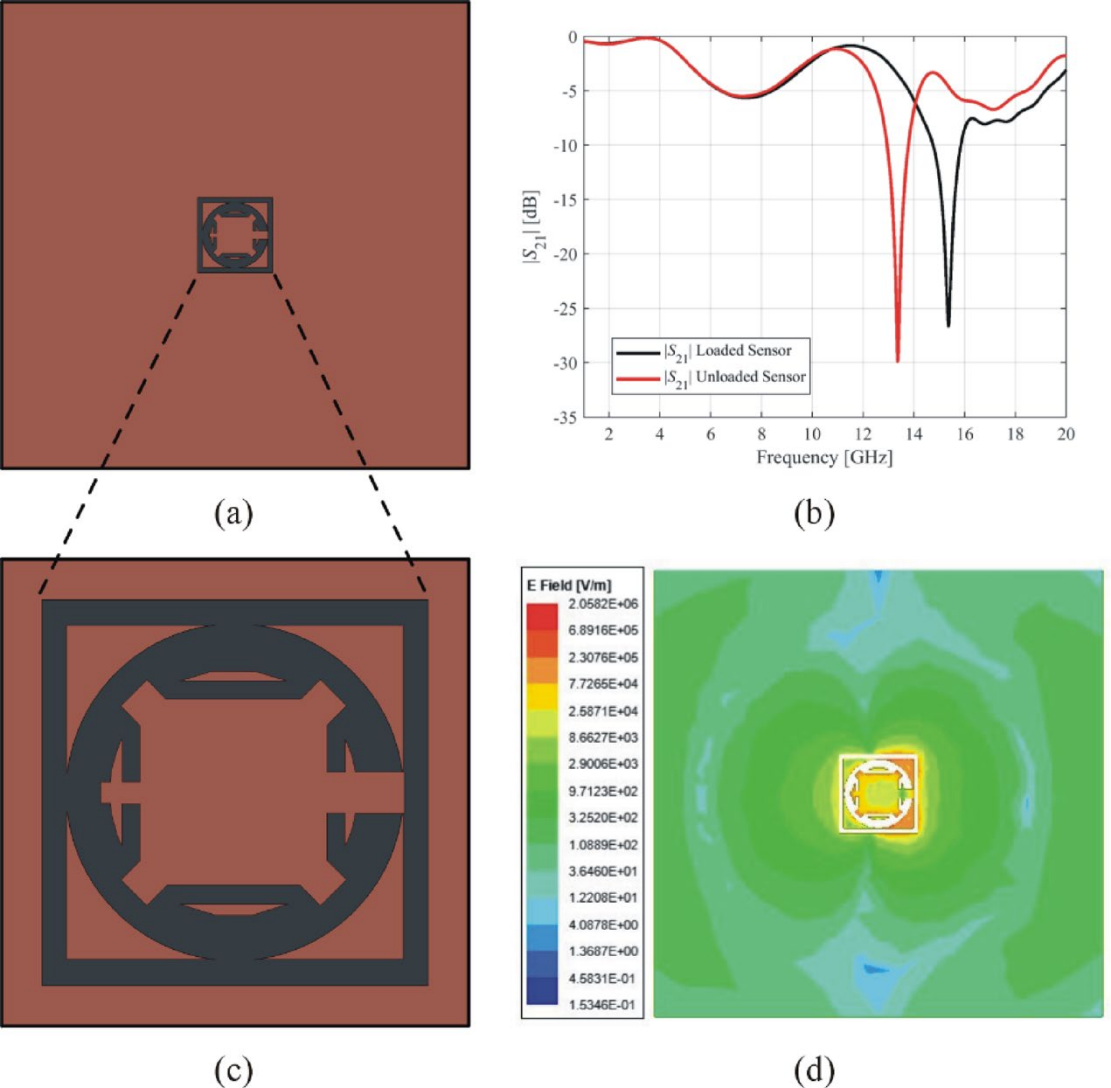
Exemplary Sensor 3 (**a**) ground layer, (**b**) transmission response, (**c**) geometry of the complementary resonator, (**d**) electric field emitted from the complementary resonator at 15.3 GHz.

The third example, illustrated in Fig. 16(a), shows the ground layer of Sensor 3. The resonant frequency of this sensor is 15.36 GHz with a notch depth of −26.64 dB, as indicated by the transmission response in Fig. 16(b). The complementary resonator’s geometry for Sensor 3 is depicted in Fig. 16(c), whereas the corresponding electric fields is illustrated in Fig. 16(d). The electric field exhibits its highest intensity near the borders of the inner square and ring of sensor 3, reaching a maximum magnitude of 2.05 × 10^6^ V/m. The loaded sensor with the MUT has a resonant frequency of 13.46 GHz with a notch depth of − 31.2 dB. This sensor exhibits a relative sensitivity of 10.30%.

**Fig. 17:**
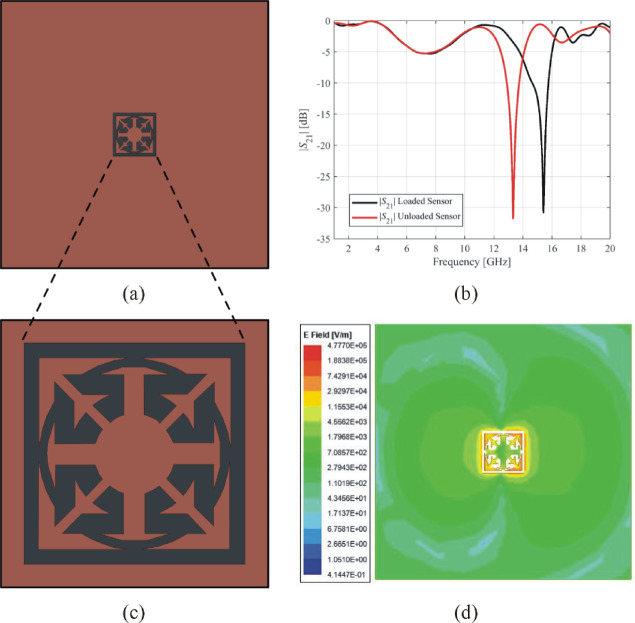
Exemplary Sensor 4 (**a**) ground layer, (**b**) transmission response, (**c**) geometry of the complementary resonator, (**d**) electric field emitted from the complementary resonator at the 15.4 GHz.

The last example, Sensor 4, has been shown in Fig. 17(a). This sensor’s notch depth is − 30.8 dB, and its resonant frequency is 15.42 GHz, as shown by the transmission response in Fig. 17(b). The geometry of the complementary resonator of Sensor 4 is shown in Fig. 17(c), and the electric field generated by this sensor is shown in Fig. 17(d). The electric field is symmetrical and exhibits a maximum magnitude of 4.77 × 10^6^ V/m. The loaded sensor with the MUT has a resonant frequency of 13.32 GHz with a notch depth of − 31.7 dB.

**Table 4:** Geometrical dimensions of the sensors generated by our approach.

Geometric variables for sensors	Sensor_1 values (mm)	Sensor_2 values (mm)	Sensor_3 values (mm)	Sensor_4 values (mm)
*as*_11_	1.82	1.81	1.04	1.09
*as*_12_	0.27	0.27	0.21	0.36
*as*_21_	1.19	1.18	1.87	1.74
*as*_22_	0.30	0.30	0.27	0.26
*ac*_11_	1.05	1.04	1.39	1.59
*ac*_12_	0.34	0.34	0.47	0.15
*ac*_21_	1.54	1.53	1.49	0.74
*ac*_22_	0.28	0.28	0.37	0.43
*cl*_1_	1.14	1.13	0.45	1.49
*cw*_1_	0.38	0.38	0.39	0.38
*cl*_2_	1.64	1.63	1.58	2.08
*cw*_2_	0.39	0.39	0.41	0.17
*sl*_1_	1.83	1.82	1.90	0.62
*sw*_1_	0.37	0.37	0.46	0.30
*sl*_2_	1.69	1.69	1.48	1.41
*sw*_2_	0.44	0.44	0.24	0.34

**Table 5:** Comparison of loaded and unloaded resonant frequencies, geometrical sizes, and sensitivity of exemplary sensors.

Exemplary Sensor	Unloaded Sensor Resonant Frequency (GHz/dB)	Loaded Sensor Resonant Frequency (GHz/dB)	External Size of Resonator (mm × mm)	Sensitivity (%)
1	17.08/-19.44	14.86/-31.08	4.19 × 4.19	10.82
2	14.90/-26.63	12.94/-26.22	4.18 × 4.18	10.95
3	15.36/-26.64	13.46/-31.22	4.28 × 4.28	10.30
4	15.42/-30.78	13.32/-31.74	4.03 × 4.03	11.34

This sensor exhibits a relative sensitivity of 11.34%. The geometric dimensions of all presented sensors can be found in Table 4 in terms of the design variable values of all their corresponding building blocks. The resonance frequencies, geometrical dimensions, and sensitivity of all the exemplary sensors are compared in Table 5. The exemplary sensors have a sensitivity exceeding 10%. Among these sensors, Sensor 4 stands out with the maximum sensitivity, which may be attributed to its compact size and symmetrical electric field. The fabrication and measurement of Sensor 4 will be carried out in the subsequent section.

## Experimental validation and benchmarking

**Fig. 18:**
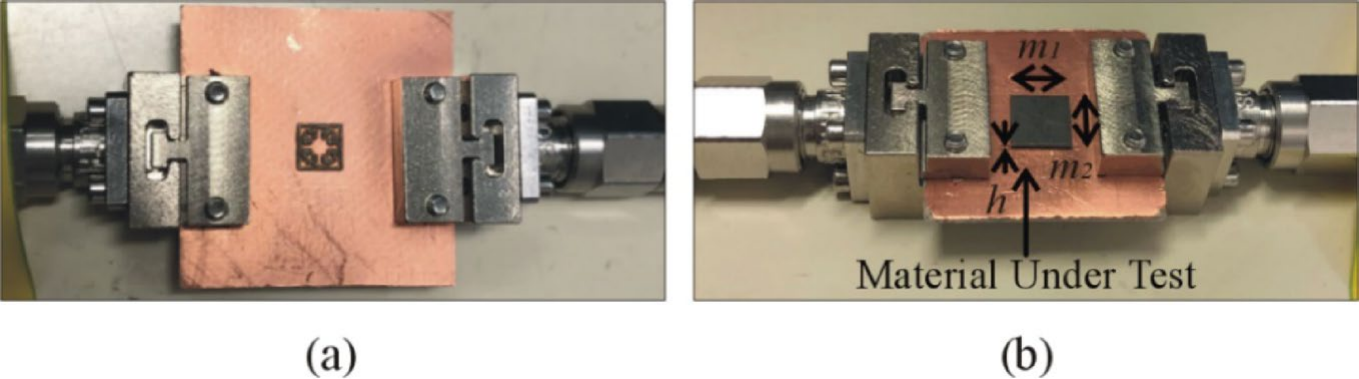
Fabricated Sensor 4: (**a**) unloaded, (**b**) loaded with material under test.

**Fig. 19:**
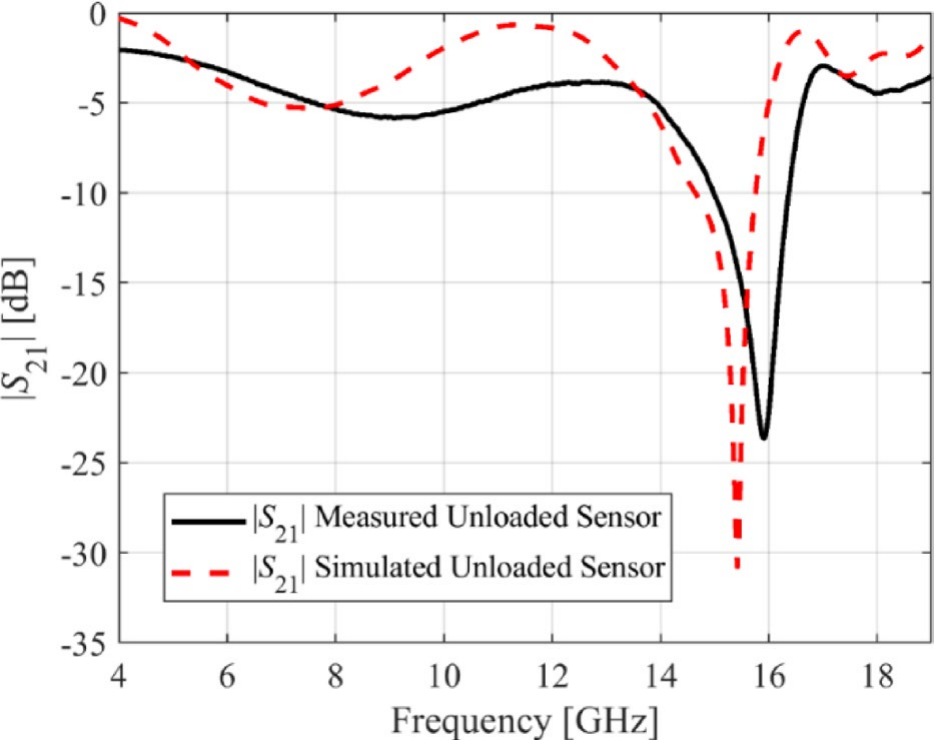
Simulated versus measured transmission coefficients (*S*_21_) for the sensor in its unloaded state.

**Fig. 20:**
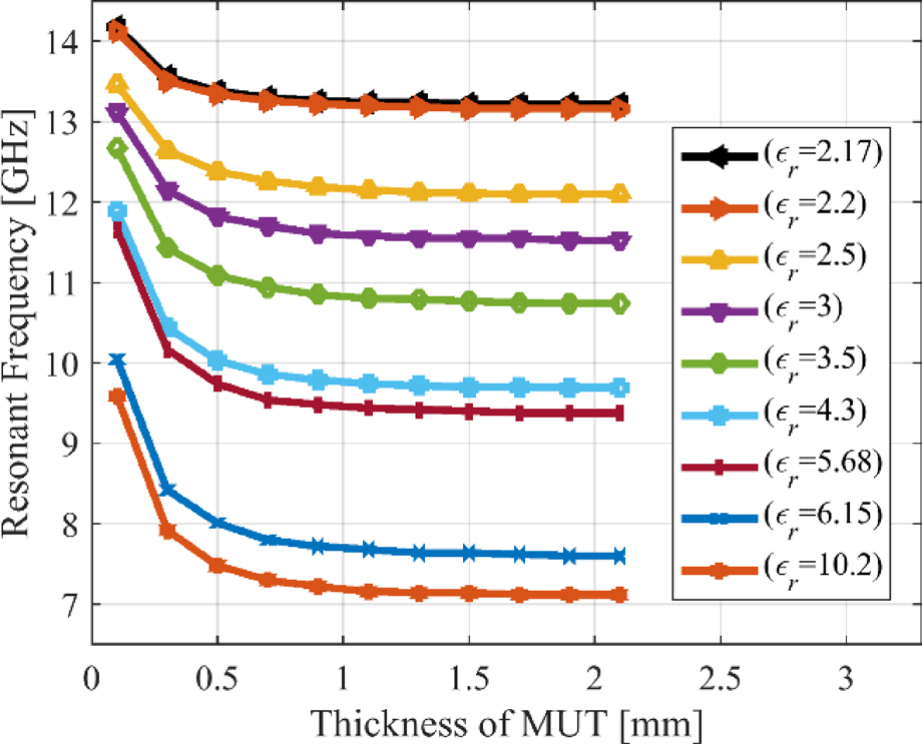
Simulated resonant frequencies of Sensor 4 due to its interaction with materials of different thicknesses (ranging from 0.1 mm to 2.1 mm) and relative permittivity values (ranging from 2.17 to 10.2).

**Fig. 21:**
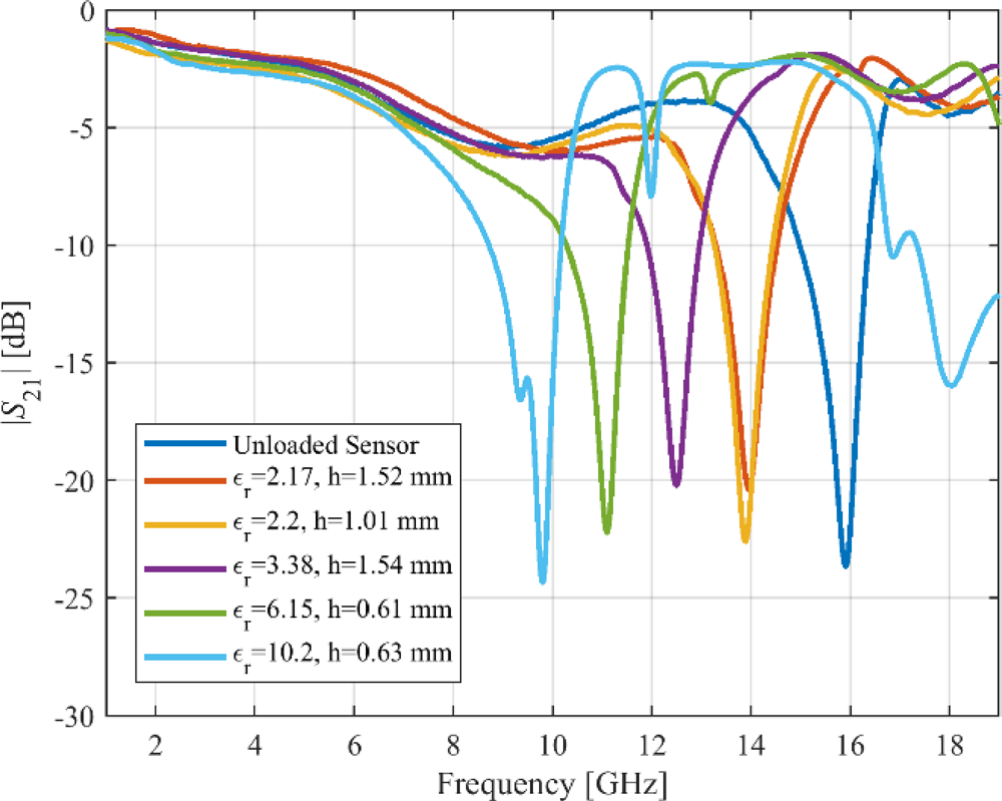
Transmission coefficients measured for the fabricated sensor when loaded with various materials undergoing testing.

For additional demonstration, this section provides a detailed description of the manufacturing specifications and calibration for Sensor 4. The LPKF Protolaser U4 has been used to fabricate the sensor on the RT5880 printed circuit board (PCB) with a dual-layered copper (Cu) plating of 17.5 μm. The LPKF ProtoLaser U4 is a laser device that uses ultraviolet light with a wavelength of 355 nm and is guided by a scanner. The laser has been purposefully engineered for the manufacturing of high-frequency PCB components. The laser beam, with a diameter of around 20 μm, allows for the fabrication of structures on the PCB with a spacing of 65 μm. This spacing consists of lines that are 50 μm wide and are separated by a distance of 15 μm. The prototype of the fabricated sensor in both loaded and unloaded states has been shown in Fig. 18. The Anritsu MS4644B (40 GHz) vector network analyzer (VNA) is used to obtain the transmission coefficients of both the loaded and unloaded sensors. Figure 19 shows the comparison between the transmission coefficient of the unloaded sensor as simulated and measured. The resonant frequency of Sensor 4 is 15.42 GHz (simulated) and 15.91 GHz (measured). The corresponding dB values for these frequencies are − 30.8 and − 23.6. The observed discrepancy of 0.49 GHz in both findings can be attributed to fabrication tolerances, which include changes in geometric factors, material properties, and roughness of the substrate’s surface. To evaluate the specific effects of MUT permittivity and thickness on the resonant frequency of the sensor, a total of nine materials under test (MUT), each with consistent dimensions of 5 × 5 mm, are positioned on the ground plane of Sensor 4. The thickness of each MUT is modified within a range of 0.1 mm to 2.1 mm, and the sensor’s transmission coefficient is simulated based on its interaction with each MUT. Figure 19 shows the outcomes obtained by altering the thickness of the MUT while maintaining a constant relative permittivity for each MUT. The resonant frequency of the sensor decreases as the thickness of the MUT increases. The impact of thickness on the resonant frequency is more significant in the range of 0.1 mm to 0.5 mm compared to the range of 0.5 mm to 1 mm. The influence of thickness diminishes gradually from 1.0 mm to 1.5 mm, and after 1.5 mm it completely ceases. The resonant frequency of the sensor is inversely related to both the permittivity and thickness of the MUT. However, the impact of permittivity is more significant, as shown in Fig. 20. The permittivity of the MUT directly affects the electrical properties of the sensor, having a greater impact on the resonant frequency than thickness. To calibrate, the fabricated sensor is loaded with different MUTs, and the transmission coefficient is measured, as shown in Fig. 21. Each MUT has a fixed size (*m*_1_ = 5 mm, *m*_2_ = 5 mm), although the relative permittivity (*ε*_*r*_ = 2.17–10.2) and thickness (*h* = 0.2–1.9) vary. The fabricated sensor exhibits a relative sensitivity of 10.69% as a result of interactions with the TLY-5 A substrate.

**Fig. 22:**
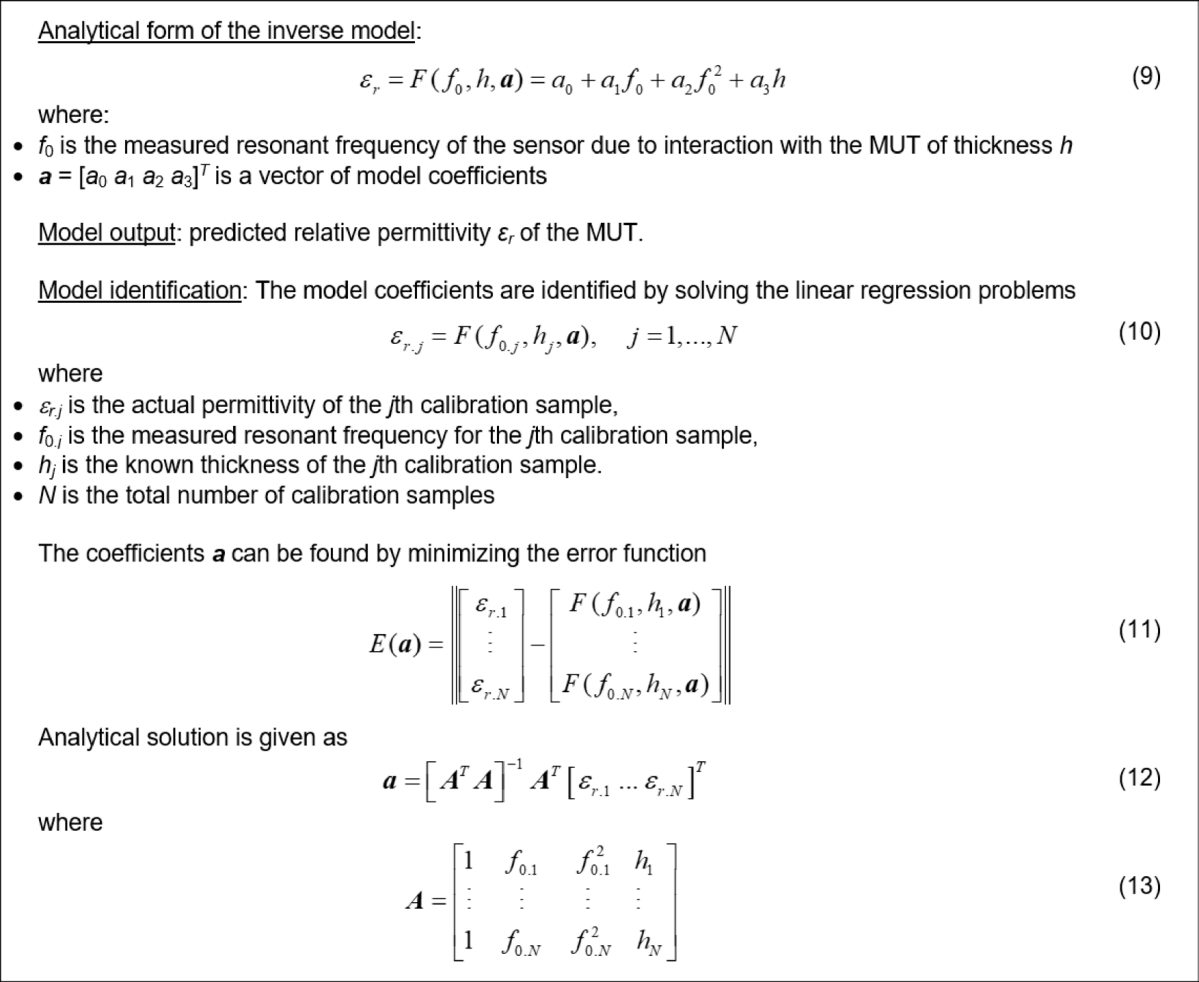
Inverse calibration model and its identification process.

By processing this information, an inverse regression model is determined to realize the sensor’s calibration. This approach enables precise estimation of the MUT’s permittivity by utilizing the actual sample thickness and the measured resonant frequency of the MUT-loaded sensor. The latter depends on both the aforementioned factors, *ε*_*r*_ and *h*, so that these variables must be used as the inputs of the calibration model. Due to the weakly nonlinear relationship between MUT’s properties and the resonant frequency, the inverse model is assumed to have a simple analytical form. This ensures that any fluctuations in the resonant frequency of the training dielectric samples, which may be caused by measurement inaccuracies, can be smoothed out during the regression process. To ensure this, the number of degrees of freedom of the inverse model must be lower than the number of data samples used to establish it. Figure 22 shows details concerning the model and its identification method.

It should be mentioned that thickness *h* is an extra degree of freedom, which defines the sample and is used as one of the input parameters of the calibration model. Consequently, as the center frequency of the loaded sensor also depends on this parameter, the proposed methodology and resulting sensors can be used to estimate the permittivity of materials of different thicknesses. This is another advantage of our technique. On the other hand, a specific sample thickness assumed at the design process (cf. Section “Unsupervised sensor design: methodology”) is necessary to compute the device’s sensitivity (maximization of which is one of the design objectives).

In this investigation, we used twelve calibration samples. Table 6 provides information about the samples’ material properties and measurement findings. Ten independent measurements were used to assess the resonant frequency; the mean and standard deviation are listed in the table. The latter is employed to calculate the measurement error. The inverse model has been identified using the procedure outlined in Fig. 22.

The obtained coefficient values are ***a*** = [48.179 − 5.545 0.164 − 0.644]^*T*^. Thus, the ultimate calibration model is given as

14 $$F({f_0},h,{\mathbf{a}})=48.179 - 5.545{f_0}+0.164f_{0}^{2} - 0.644h$$

**Fig. 23:**
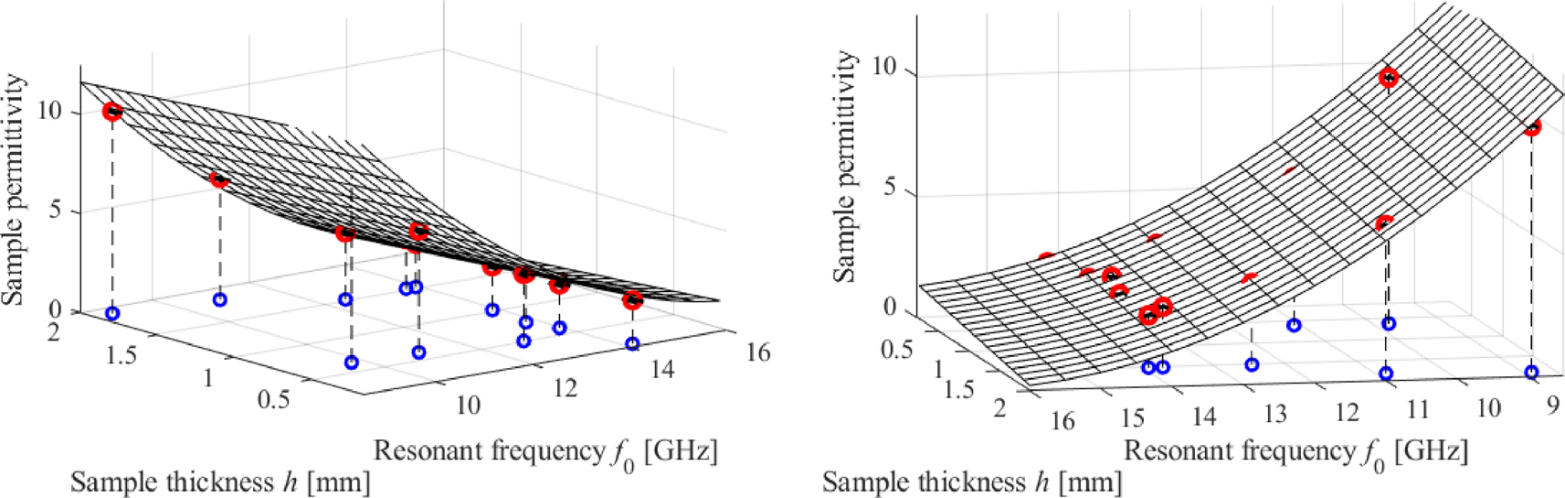
Inverse model (1) identified to enable calibration of the fabricated microstrip sensor. The input parameters include the center frequency *f*_0_ and the sample’s thickness *h*. In the picture, red circles mark the calibration samples; blue circles represent samples’ projections onto the *f*_0_-*h* space. Standard deviation of *f*_0_, obtained from ten independent measurements, are marked using the horizontal bars. To improve clarity, two different viewpoints are given.

Figure 23 provides a visualization of the inverse model with superimposed calibration samples and the associated error bars.

To validate the calibration technique and assess the properties of the sensor, the inverse model was utilized to forecast the relative permittivity of thirteen testing MUT samples, as outlined in Table 7. The inverse model demonstrates exceptional predictive capabilities, as can be clearly shown. The average relative error in forecasting the MUT’s permittivity is approximately 3%, while the highest error is approximately 7%.

By utilizing the estimated maximum error of measuring the resonant frequency *df*_0_ (which is determined as 0.2 GHz based on the data in Table 6), along with the sensitivity *∂F*/*∂f*_0_ of the inverse model concerning the center frequency at the frequency of measurement and the MUT’s thickness *h*, one can compute the maximum estimated prediction error, which is

15 $$d\varepsilon =\frac{{\partial F({f_0},h,{\mathbf{a}})}}{{\partial {f_0}}}d{f_0}$$

**Fig. 24:**
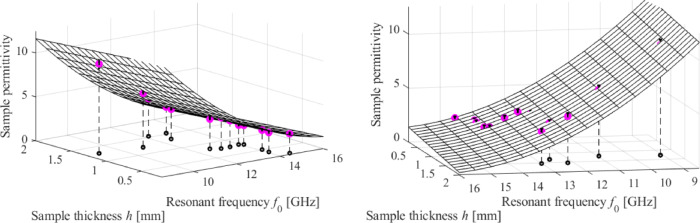
Inverse model validation. Circles indicate the test samples. The estimated permittivity prediction error is represented using vertical bars. The calibration model over the *f*_0_-*h* space is shown as a surface. Small circles are projections of the testing samples onto the space of *f*_0_-*h* space. To enhance clarity, two distinct viewing angles are shown.

**Table 6:**
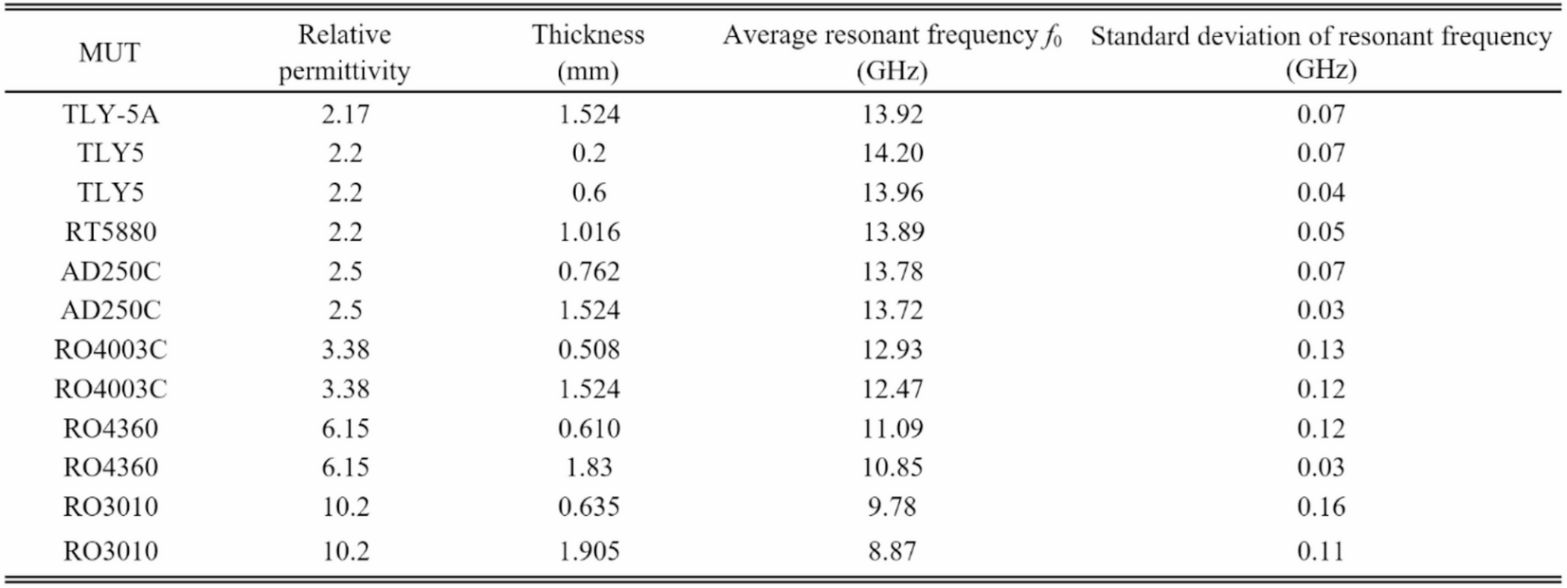
Material parameters and measured data for calibration samples.

**Table 7:**
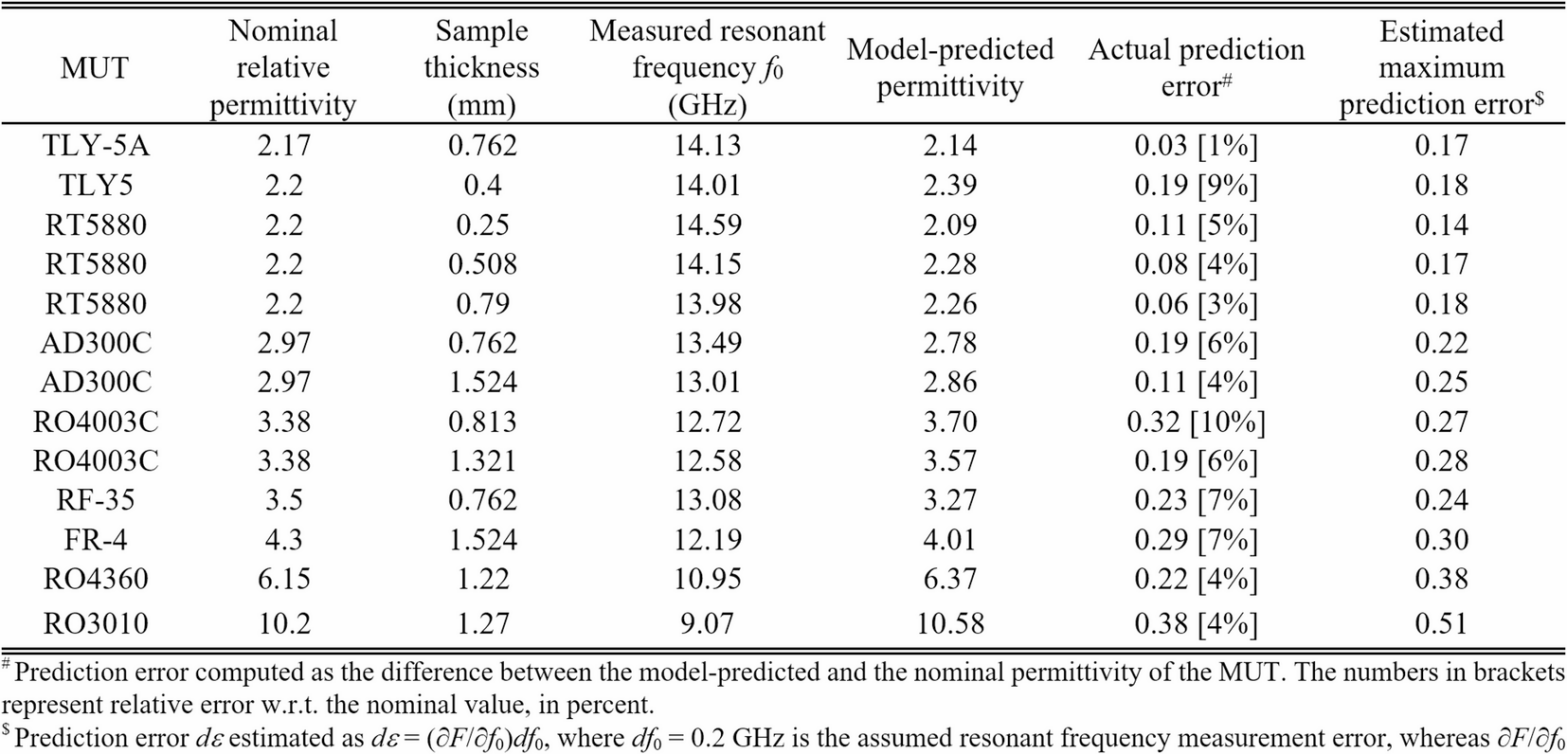
Sensor validation: model-predicted versus actual sample permittivity.

**Table 8:** A comparison with the latest microwave sensors incorporating complementary resonators.

Ref.	Design Technology	Resonant Frequency (GHz)	Measured Permittivity Range	Calibration Model	Maximum Relative Sensitivity (%)
^50^	Manual Intervention	7.20	2.52–4.47	Least Square	5.21
^51^	Manual Intervention	14.45	2.2–5.5	Inverse Regression	5.41
^52^	Manual Intervention	14.62	2.2–4.3	Inverse Regression	7.01
^53^	Manual Intervention	8.5	2.46–2.58	Curve Fitting	7.25
^27^	Manual Intervention	12.6/16.2	2.38–2.45	Curve Fitting	7.4/9.9
^54^	Manual Intervention	17.39	2.2–10.2	Inverse Regression	8.05
This work	Artificial Intelligence	15.91	2.2–10.2	Inverse Regression	10.69

The values of *d*_*ε*_ for all MUTs can be found in the right-hand-side column of Table 7. Figure 24 displays the calibration model predictions for the testing samples, overlaid over the inverse model surface, along with the appropriate error bars. The fabricated sensor and its calibration technique allow for reliable predictions of material parameters throughout a wide range of relative permittivity, ranging from around two to over ten.

Table 8 presents a detailed comparison between selected state-of-the-art complementary resonator-based sensors and the fabricated sensor in terms of their sensitivity and the range of permittivity measurements. The data supplied in Table 8 corroborates that the electrical performance of the sensor presented in this section and developed using AI-based approach proposed in this study is highly competitive to the benchmark sensors reported in the literature that operate in similar frequency ranges.

At this point, it is appropriate to summarize the advantages of the proposed unsupervised design technique and juxtapose it against conventional sensor design methodologies:

- In practice, conventional sensor design involves several steps, which include topology evolution decided upon using experience, trial and error, and repetitive parametric studies, eventually followed by more or less rigorous optimization. A process like that typically takes a considerable amount of time (up to several weeks) and requires regular expert interaction. The proposed approach is unsupervised and fully automated, and, eventually, much faster.
- Conventional design does not explicitly account for sensitivity, which is the main performance indicator. Typically, design procedures attempt to obtain a specific location of the resonance and the notch depth. Our technique explicitly maximizes the sensitivity.
- Conventional methods are limited in terms of the number of architectures that can be explored. Also, they are unable to handle more than a few parameters, which is not the case in our method.

As indicated in Table 8, our technique leads to considerably better results in terms of sensor’s sensitivity (close to eleven, compared to between five and nine for the benchmark structures.

## Conclusion

In this paper, we have presented an innovative approach to the unsupervised design and optimization of resonator-based microstrip sensors using artificial intelligence (AI) methods. Our method employs fundamental building elements such as circular and square resonators, stubs, and slots, which are integrated into geometrically involved designs using unit cell relocation and size adjustments, as well as utilization of Boolean transformations. The design process, driven solely by specifications, harnesses evolutionary algorithms and gradient-based optimizers to explicitly enhance sensor sensitivity and to ensure the attainment of desired operating frequencies. This research aimed to address the challenge of designing high-performance sensors for dielectric material characterization without the need for manual intervention. By employing AI-driven techniques, we achieved significant advancements in both sensitivity enhancement and frequency optimization. Through extensive validation, calibration, and experimentation, we demonstrated the effectiveness of our approach in designing sensors capable of accurately characterizing dielectric samples across a wide range of permittivity and thickness. Our findings represent a significant contribution to the field of sensor design and optimization. The AI-generated sensors exhibited superior performance compared to state-of-the-art designs reported in the literature, showcasing the potential of AI methods in revolutionizing sensor technology. The proposed methodology faced challenges in incorporating the complex levels of freedom necessary for generating structures like the dual-ring CSRR within the limitations of our current approach. In the future, we aim to explore further and enhance our optimization technique to overcome these limitations. Another planned extension will be to account for the fabrication tolerances by adding another design goal, which is the tolerance-induced variability of the center frequency (quantified, for example, using its statistical moments as a function of the probability density function describing the fabrication-process-related manufacturing inaccuracies). In practice, the local objective function (4) may be augmented by another penalty factor computing, e.g., the estimated standard deviation of the center frequency. The estimation itself may be obtained using a simplified linear model of the sensor’s frequency characteristics (available anyway due to being required by the trust-region algorithm performing fine tuning). This extension will be considered as part of future work.

## Data Availability

The datasets used and/or analyzed during the current study are available from the corresponding author on reasonable request.

## References

[1] Wu, W. J., Zhao, W. S. & Wang, W. A novel differential microwave sensor based on reflective-mode phase variation of stepped-impedance transmission lines for extracting permittivity of dielectric materials. *IEEE Sens. J.* **24** (3), 2746–2757 (2024).

[2] Shah, A. et al. Unlocking conformal microwave split ring resonant sensors for in-flight ice sensing. *IEEE Trans. Aerosp. Electron. Syst.* **60** (1), 525–536 (2024).

[3] Paz Silva, L. A., Brito, F. A., Filho & de Andrade, H. D. Soil moisture monitoring system based on metamaterial-inspired microwave sensor for precision agriculture applications. *IEEE Sens. J.* **23** (19), 23713–23720 (2023).

[4] Sun, B. M., Kenney, R. H., Yeary, M. B., Sigmarsson, H. H. & McDaniel, J. W. Reduced navigation error using a multi-sensor fusion technique and its application in synthetic aperture radar. *IEEE J. Microwaves*. **4** (1), 86–100 (2024).

[5] Raju, R. & Bridges, G. E. A compact wireless passive harmonic sensor for packaged food quality monitoring. *IEEE Trans. Microw. Theory Tech.* **70** (4), 2389–2397 (2022).

[6] Dey, U. & Hesselbarth, J. Subwavelength particle spectroscopy by measurements of electromagnetic scattering at millimeter-wave frequency. *IEEE Trans. Microw. Theory Tech.* **70** (1), 699–710 (2022).

[7] Wang, D. et al. Measurement of solid concentration in gas–solid flows using a microwave resonant cavity sensor, *IEEE Trans. Instrum. Meas.* **73**, 7501009, 1–9, (2024).

[8] Masi, A., Brizim, D. & Monorchio, A. Millimetric inclusion detection through a contactless microwave spiral sensor for biomedical applications. *IEEE Sens. J.* **23** (12), 12796–12807 (2023).

[9] Chavoshi, M., Martinic, M., Nauwelaers, B., Markovic, T. & Schreurs, D. Design of uncoupled and cascaded array of resonant microwave sensors for dielectric characterization of liquids. *IEEE Trans. Microw. Theory Tech.* **71** (4), 1687–1695 (2023).

[10] Song, X., Yan, S. & Chen, J. A design of metamaterial inspired resonator for multifrequency fluidic sensors. *IEEE Microw. Wirel. Tech. Lett.* **33** (1), 94–97 (2023).

[11] Wu, W. J. & Zhao, W. S. A microwave sensor based on frequency-locked loop and multiple complementary split-ring resonators for retrieving complex permittivity of liquid samples. *IEEE Sens. J.* **23** (24), 30345–30359 (2023).

[12] Niksan, O., Jain, M. C., Shah, A. & Zarifi, M. H. A nonintrusive flow rate sensor based on microwave split-ring resonators and thermal modulation. *IEEE Trans. Microw. Theory Techn*. **70** (3), 1954–1963 (2022).

[13] Lv, B. et al. Permittivity and concentration measurements based on coplanar waveguide and split ring resonator sensor. *IEEE Sens. J.* **24** (4), 5122–5131 (2024).

[14] Moqadam, A. N. & Kazemi, R. High-resolution imaging of narrow bone fractures with a novel microwave transceiver sensor utilizing dual-polarized RIS and SRR array antennas. *IEEE Sens. J.* **23** (24), 4424–4431 (2024).

[15] Bazgir, M. & Sheikhi, A. High Q-factor compact permittivity sensor based on coupled SRR-ELC metamaterial element and metasurfaces shield. *IEEE Sens. J.* **24** (4), 4424–4431 (2024).

[16] Han, H. et al. Remote sensing image classification based on multi-spectral cross-sensor super-resolution combined with texture features: a case study in the Liaohe planting area. *IEEE Access.* **12**, 16830–16843 (2024).

[17] Wang, Z., Yang, X., Zhou, X., Su, P. & Wang, J. A flexible sensor Tag for surface crack detection of curved film-coated metals. *IEEE Sens. J.* **22** (6), 5662–5668 (2022).

[18] Keshavarz, R., Lipman, J., Schreurs, D. M. M. P. & Shariati, N. Highly sensitive differential microwave sensor for soil moisture measurement. *IEEE Sens. J.* **21** (24), 5662–5668 (2022).

[19] Abdolrazzaghi, M., Katchinskiy, N., Elezzabi, A. Y., Light, P. E. & Daneshmand, M. Noninvasive glucose sensing in aqueous solutions using an active split-ring resonator. *IEEE Sens. J.* **21** (17), 18742–18755 (2021).

[20] Abdolrazzaghi, M., Zarifi, M. H., Pedrycz, W. & Daneshmand, M. Robust ultra-high resolution microwave planar sensor using fuzzy neural network approach. *IEEE Sens. J.* **17** (2), 323–332 (2017).

[21] Kazemi, N. & Musilek, P. Resolution enhancement of microwave sensors using super-resolution generative adversarial network. *Expert Syst. Appl.* **213** (119252), 1–14 (2023).

[22] Kazemi, N., Abdolrazzaghi, M. & Musilek, P. Comparative analysis of machine learning techniques for temperature compensation in microwave sensors. *IEEE Trans. Microw. Theory Techn*. **69** (9), 4223–4236 (2021).

[23] Mosavirik, T., Nayyeri, V., Hashemi, M., Soleimani, M. & Ramahi, O. M. Direct permittivity reconstruction from power measurements using a machine learning aided method. *IEEE Trans. Microw. Theory Techn*. **71** (10), 4437–4448 (2023).

[24] Abdolrazzaghi, M., Kazemi, N., Nayyeri, V. & Martin, F. AI-assisted ultra-high-sensitivity/resolution active-coupled CSRR-based sensor with embedded selectivity. *Sensors* **23** (13), 1–20 (2023). 10.3390/s23136236 PMC10347157 37448086

[25] Albishi, A. M., Badawe, M. K. E., Nayyeri, V. & Ramahi, O. M. Enhancing the sensitivity of dielectric sensors with multiple coupled complementary split-ring resonators. *IEEE Trans. Microw. Theory Tech.* **68** (10), 4340–4347 (2020).

[26] Ansari, M. A. H., Jha, A. K., Akhter, Z. & Akhtar, M. J. Multi-band RF planar sensor using complementary split ring resonator for testing of dielectric materials. *IEEE Sens. J.* **18** (16), 6596–6606 (2018).

[27] Zhang, X., Ruan, C. & Cao, Y. A dual-mode microwave sensor for edible oil characterization using magnetic-LC resonators. *Sens Actuators A: Phys*, **333**, 113275, (2022).

[28] Koziel, S. & Haq, T. Computationally-efficient statistical design and yield optimization of resonator-based notch filters by feature-based surrogates. *Sc. Rep.* **13**, 14823 (2023). 10.1038/s41598-023-42056-7 PMC10491762 37684301

[29] Haq, T. & Koziel, S. Novel complementary resonator for dielectric characterization of substrates based on permittivity and thickness. *IEEE Sens. J.* **24** (1), 195–203 (2023). 10.3390/s23229138 PMC10674548 38005525

[30] Liu, P., Chen, L. & Chen, Z. N. Prior-knowledge-guided deep-learning-enabled synthesis for broadband and large phase shift range metacells in Metalens antenna. *IEEE Trans. Ant Propag.* **70** (7), 5024–5034 (2022).

[31] Ohira, M., Ban, H. & Ueba, M. Evolutionary generation of subwavelength planar element loaded monopole antenna. *IEEE Ant Wirel. Propag. Lett.* **10**, 1559–1562 (2011).

[32] Arianos, S. et al. Application of evolutionary algorithms in the design of compact multi-band antennas, *IEEE Int. Symp. Ant. Propag.*, pp. 1–2, (2012).

[33] Jiang, F., Chiu, C. Y., Shen, S., Cheng, Q. S. & Murch, R. Pixel antenna optimization using N-port characteristic mode analysis. *IEEE Trans. Ant Propag.* **68** (5), 3336–3347 (2020).

[34] Soltani, S., Lotfi, P. & Murch, R. D. Design and optimization of multiport pixel antennas. *IEEE Trans. Ant Propag.* **66** (4), 2049–2054 (2018).

[35] Lotfi, P., Soltani, S. & Murch, R. D. Printed endfire beam-steerable pixel antenna. *IEEE Trans. Ant Propag.* **65** (8), 3913–3923 (2017).

[36] Jiang, F. et al. Pixel antenna optimization based on perturbation sensitivity analysis. *IEEE Trans. Ant Propag.* **70** (1), 472–486 (2022).

[37] Zhu, S. H., Yang, X. S., Wang, J. & Wang, B. Z. Design of MIMO antenna isolation structure based on a hybrid topology optimization method. *IEEE Trans. Ant Propag.* **67** (10), 6298–6307 (2019).

[38] Wang, J., Yang, X. S., Ding, X. & Wang, B. Z. Topology optimization of conical-beam antennas exploiting rotational symmetry. *IEEE Trans. Ant Propag.* **66** (5), 2254–2261 (2018).

[39] Wang, J., Yang, X. S., Ding, X. & Wang, B. Z. Antenna radiation characteristics optimization by a hybrid topological method. *IEEE Trans. Ant Propag.* **65** (6), 2843–2854 (2017).

[40] Naseri, P. & Hum, S. V. A generative machine learning-based approach for inverse design of multilayer metasurfaces. *IEEE Trans. Ant Propag.* **69** (9), 5725–5739 (2021).

[41] Hassan, E., Noreland, D., Augustine, R., Wadbro, E. & Berggren, M. Topology optimization of planar antennas for wideband near-field coupling. *IEEE Trans. Ant Propag.* **63** (9), 4208–4213 (2015).

[42] Koziel, S. et al. On unsupervised artificial intelligence-assisted design of antennas for high-performance planar devices, *Electronics*. **12** (16), 3462 (2023).

[43] Microwave Studio, C. S. T. ver. Dassault Systemes, France, (2021).

[44] Matlab MathWorks Inc., Natick, MA, USA, (2021).

[45] Michalewicz, Z. *Genetic algorithms + data Structures = evolution Programs* (Springer, 1996).

[46] Blickle, T. & Thiele, L. A comparison of selection schemes used in evolutionary algorithms. *Evol. Comp.* **4** (4), 361–394 (1996).

[47] Conn, A. R., Gould, N. I. M. & Toint, P. L. *Trust Region Methods* (MPS-SIAM Series on Optimization, 2000).

[48] Levy, H. & Lessman, F. *Finite Difference Equations* (Dover Publications Inc., 1992).

[49] Nocedal, J. & Wright, S. J. *Numerical Optimization* 2nd Edn (Springer, 2006).

[50] Armghan, A. Complementary metaresonator sensor with dual Notch resonance for evaluation of vegetable oils in C and X bands. *Appl. Sci.* **11** (12), 5734 (2021).

[51] Haq, T. & Koziel, S. Inverse modeling and optimization of CSRR-based microwave sensors for industrial applications. *IEEE Trans. Microw. Theory Techn*. **70** (11), 4796–4804 (2022).

[52] Haq, T. & Koziel, S. Rapid design optimization and calibration of microwave sensors based on equivalent complementary resonators for high sensitivity and low fabrication tolerance. *Sensors* **23** (2), 1044 (2023). 36679841 10.3390/s23021044 PMC9860940

[53] Zhang, X., Ruan, C., Wang, W. & Cao, Y. Submersible high sensitivity microwave sensor for edible oil detection and quality analysis. *IEEE Sens. J.* **21** (12), 13230–13238 (2021).

[54] Haq, T. & Koziel, S. Novel complementary multiple concentric split ring resonator for reliable characterization of dielectric substrates with high sensitivity. *IEEE Sens. J.* **24** (10), 16233–16241 (2024).

